# Fighting Antimicrobial Resistance: Insights on How the *Staphylococcus aureus* NorA Efflux Pump Recognizes 2-Phenylquinoline Inhibitors by Supervised Molecular Dynamics (SuMD) and Molecular Docking Simulations

**DOI:** 10.1021/acs.jcim.3c00516

**Published:** 2023-07-29

**Authors:** Deborah Palazzotti, Tommaso Felicetti, Stefano Sabatini, Stefano Moro, Maria Letizia Barreca, Mattia Sturlese, Andrea Astolfi

**Affiliations:** † Department of Pharmaceutical Sciences, Department of Excellence 2018−2022, University of Perugia, Via del Liceo, 1, 06123 Perugia, Italy; ‡ Molecular Modeling Section (MMS), Department of Pharmaceutical and Pharmacological Sciences, University of Padova, via Marzolo 5, 35131 Padova, Italy

## Abstract

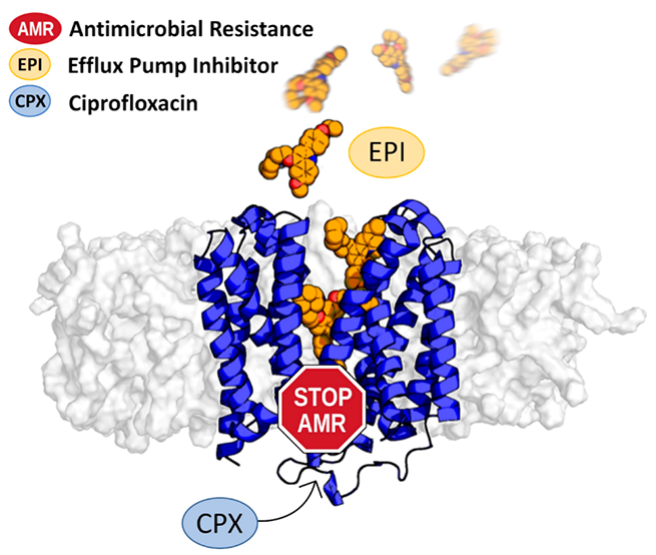

The superbug *Staphylococcus aureus* (*S. aureus*) exhibits several resistance mechanisms, including efflux pumps, that strongly contribute to antimicrobial resistance. In particular, the NorA efflux pump activity is associated with *S. aureus* resistance to fluoroquinolone antibiotics (*e.g*., ciprofloxacin) by promoting their active extrusion from cells. Thus, since efflux pump inhibitors (EPIs) are able to increase antibiotic concentrations in bacteria as well as restore their susceptibility to these agents, they represent a promising strategy to counteract bacterial resistance. Additionally, the very recent release of two NorA efflux pump cryo-electron microscopy (cryo-EM) structures in complex with synthetic antigen-binding fragments (Fabs) represents a real breakthrough in the study of *S. aureus* antibiotic resistance. In this scenario, supervised molecular dynamics (SuMD) and molecular docking experiments were combined to investigate for the first time the molecular mechanisms driving the interaction between NorA and efflux pump inhibitors (EPIs), with the ultimate goal of elucidating how the NorA efflux pump recognizes its inhibitors. The findings provide insights into the dynamic NorA-EPI intermolecular interactions and lay the groundwork for future drug discovery efforts aimed at the identification of novel molecules to fight antimicrobial resistance.

## 1 Introduction

The use of large quantities of antibiotics to control bacterial infections has created unprecedented conditions for the development of antimicrobial resistance (AMR).^1−3^ Microorganisms can employ five main mechanisms to drive AMR named: (i) inactivation of the antibiotic, (ii) target modification, (iii) reduced penetration of the antibiotic, (iv) overexpression of efflux pumps, and (v) biofilm formation.^4,5^ Notably, the overexpression of efflux pumps represents a major mechanism of clinical resistance leading to an increase of drug extrusion that, coupled with the promiscuous activity of the efflux pumps, can result in superbugs potentially untreatable with conventional therapies.^6,7^

Since bacterial efflux pumps play a crucial role in the development of AMR both in animals and plants, the identification of efflux pump inhibitors (EPIs) and their coadministration with antibiotic agents has the potential to restore the antibacterial activity of drugs^8−10^ or agrochemicals^11−13^ to their original levels. In this scenario, *Staphylococcus aureus* (*S. aureus*) stands out as the most dangerous superbug among Gram-positive organisms,^14−16^ with the transmembrane protein NorA being involved in resistance mechanisms against a plethora of structurally unrelated natural and synthetic compounds (*e.g*., quaternary ammonium compounds and antiseptics, phenothiazines and thioxanthenes, totarol, ferruginol, carnosic acid, ethidium bromide (EtBr), tetraphenylphosphonium, rhodamine, acridine, and biocides).^17,18^ In particular, NorA overexpression is associated with quinolone and fluoroquinolone (*e.g*., ciprofloxacin) resistance.^18^

As a major facilitator superfamily (MFS) member, the NorA single polypeptide consists of 12 hydrophobic transmembrane α-helices (TMH) with amino (N-) terminal and carboxyl (C-) terminal domains, arranged in a pseudo-2-fold symmetry. Both N- and C-termini are placed on the cytoplasmic side of the membrane.^19^ NorA can exist in two different conformations known as the “outward conformation” (C_out_) with an opening toward the extracellular side and the “inward conformation” (C_in_) with an opening toward the cytoplasmic side. The mechanism of drug extrusion is poorly understood; nevertheless, it is known that NorA, which acts as a drug/H^+^ antiporter, couples the entry of a proton into the extrusion of the drug from the cell.

**Figure 1:**
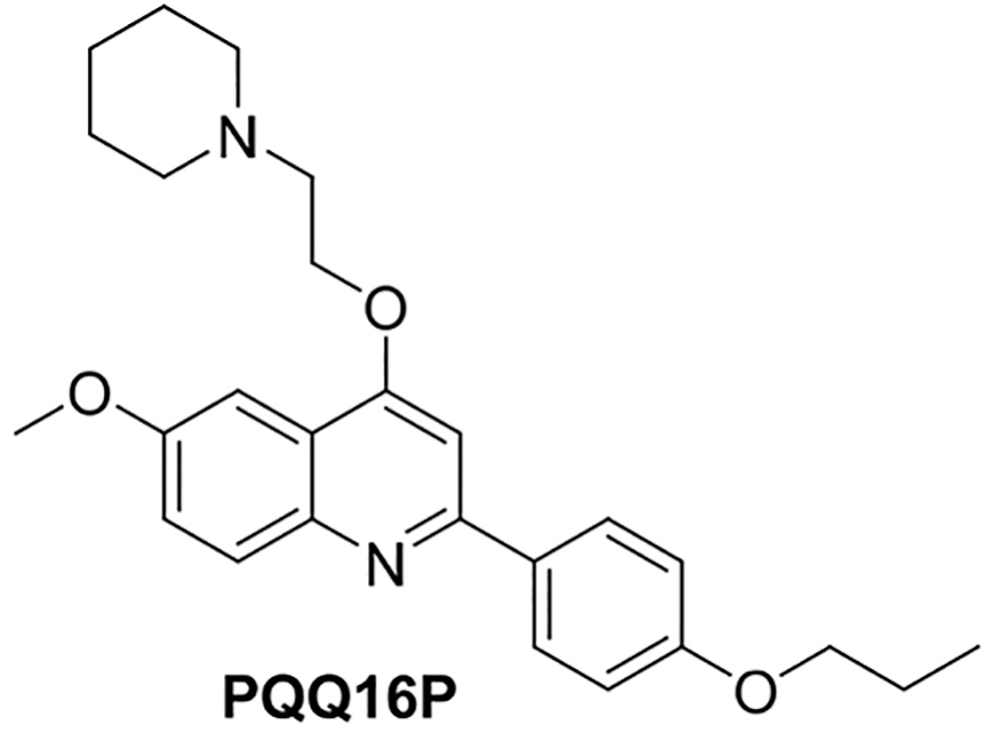
2D Chemical Structure of **PQQ16P**.

Given the crucial involvement of NorA in *S. aureus* efflux-mediated antimicrobial resistance, many research groups along the years have been working on the identification of new NorA EPIs by using different approaches such as isolation of bioactive compounds from natural sources,^20,21^ drug repurposing strategies, or classical medicinal chemistry efforts.^22^ In this context, several compounds belonging to different chemical families are known (*e.g.,* benzothiazine,^23^ benzothiophene,^24^ boronic acid,^25^ indole,^26^ piperine,^27^ naphthyridine,^28^ quinoline,^29^ and quinolone^29^ analogues). As a research group, we focused our efforts on the identification and exploration of quinoline-like derivatives as potent NorA EPIs.^30−34^ Within this chemical series, **PQQ16P** (see Figure 1) resulted in one of the most promising NorA EPIs so far reported due to its strong ability to inhibit EtBr efflux (97.6% when using the SA-1199B strain overexpressing *norA* and harboring a GrlA mutation) and a marked ability to restore the ciprofloxacin antibacterial effect in resistant *S. aureus* strains. Indeed, **PQQ16P** at 0.78 μg/mL was able to reduce the ciprofloxacin MIC by 8-fold (from 10 to 1.25 μg/mL) against SA-1199B, while not showing any significant synergistic effect with ciprofloxacin when tested against *S. aureus* wild-type strains (ATCC25923 and SA-1199). This specific effect on the NorA activity by **PQQ16P** was further confirmed by membrane polarization assays on SA-1199B (no significant depolarization was observed in the presence of **PQQ16P**) and checkerboard assays with ciprofloxacin using the parent *S. aureus* strains (SA-K1902 and SA-K2378), different from each other for the absence or overexpression of *norA*, respectively. As expected, **PQQ16P** showed synergistic activity with ciprofloxacin only against SA-K2378, completely restoring its antibacterial activity. Further profiling of **PQQ16P** displayed a promising metabolic stability and a low toxicity toward human cell lines (CC_50_ = 42 μg/mL on HepG2 and >100 μg/mL on THP-1), making it a good candidate for more in-depth biological studies.^35^

However, the lack of structural information about the NorA protein has strongly, so far, hampered structure-based discovery efforts in this field. Additionally, the structural diversity of NorA EPIs, coupled with the lack of sufficient structure–activity relationship information, made it difficult to get clues for the rational design of new EPI candidates.

Generally, in the absence of any structural data for the investigated protein, such as that obtained from X-ray crystallography or NMR spectroscopic methods, it may be possible to build 3D protein models or use closely related protein structures as surrogate models.^36,37^ Indeed, in a previous study, we generated and exploited a homology model of NorA deciphering for the first time the molecular recognition pathway between this efflux pump and the substrate ciprofloxacin.^34^ This work provided intriguing insights into the protein sites explored during the ligand trajectory prior to its extrusion toward the extracellular side.

In March 2022, the first experimental 3D structures of efflux pump NorA from *S. aureus* were made available. In particular, two cryo-EM NorA protein structures (C_out_ conformation) in complex with Fab fragments (PDB IDs 7LO8 and 7LO7) were released.^19^ Notably, this study demonstrated that the cocrystallized Fab portions acted as EPIs. These high-resolution structures of NorA can contribute significantly to a better understanding of how this pump recognizes its substrates/inhibitors as well as to the design and development of more target-specific and effective EPIs.

Indeed, here, these structural data were exploited for the first time to decipher the molecular event underlying EPI-NorA recognition. To achieve the set goal, we combined the supervised molecular dynamics (SuMD) method with molecular docking experiments.

Specifically, SuMD is an enhanced sampling technique able to sample the mutual conformational changes occurring between the protein and ligand during ligand-binding events.^38^ SuMD has proved to be able to provide useful information about the recognition event sampling metastable binding events proceeding the final bound state.^39^ In addition, SuMD has demonstrated the ability to identify the native binding state in many different systems^38,40−42^ and also in a blind challenge.^43^ The approach consists of a succession of consecutive short unbiased MD classical simulations (600 ps) supervised by a tabu-like algorithm that decides if each short simulation aimed to enhance the recognition pathway from the unbound state. It is worth noting that this computational method allows a reduction of the sampling of binding events to a nanosecond time scale, without introducing any energetic bias to the simulation and simulating the explicit desolvation of the binding process.^44^

In the present work, we specifically investigated the complex interplay between NorA and in-house 2-phenylquinoline NorA EPIs. In particular, SuMD simulations were performed to explore at the molecular level the possible recognition pathway and interactions established between the representative quinoline derivative **PQQ16P** and the efflux pump. Molecular docking experiments were then run to determine whether other active analogues shared the predicted binding mode for **PQQ16P**.

## 2 Methods

All simulations were performed on a hybrid CPU/GPU cluster provided by a 24 NVIDIA graphics cards, whose models include GTX 1080ti to RTX 3090ti. MD and SuMD simulations adopted ACEMD (vers, 2018.11.26)^45^ as an MD engine. The protocol based on the CHARMM36/CHARMM general force field (CGenFF)^46^ combinations was adopted for transmembrane systems.

### 2.1 Protein Preparation

The cryo-EM conformation of NorA with the best resolution (PDB ID: 7LO8, 3.16 Å) was downloaded from Protein Data Bank (PDB)^47^ and was selected for our modeling studies. First, the NorA–Fab36 complex was prepared using Schrödinger’s Protein Preparation Wizard^48^ to obtain satisfactory starting structures for modeling studies. The complex was preprocessed as follows: (i) hydrogen atoms were added and bond orders were assigned to amino acid residues and Fab36; (ii) the missing side chains and the missed residues were filled; (iii) the protein was capped with acetyl (ACE) and *N*-methylamine (NMA) groups; (iv) all water molecules were deleted, and (v) Epik^49^ was used to predict ionization and tautomeric states for the ligand (pH = 7.0 ± 2). Then, the H-bond network of the complex was optimized using PROPKA for the assignment of the residue protonation states (pH = 7.0), and finally, the complex was submitted to a restrained minimization (OPLS3 force field) which was stopped when the RMSD of heavy atoms reached 0.30 Å. The cytoplasmatic missing loop (184–197) was filled in the complex using the homology modeling tool function implemented in the Prime package.^50^ Last, the rebuilt loop was refined using the refine loops functionality, applying as “serial loop sampling” the ultra extended mode, as suggested for loops with 10 or more residues.

### 2.2 Ligand Preparation

The active and inactive sets were constructed using in-house compounds (i) experimentally tested for their capacity to inhibit EtBr extrusion mediated by NorA and (ii) having an FTreeS similarity^51^ to **PQQ16P** greater than 0.7. The investigate inhibitor **PQQ16P** and **1**–**102** were drawn using Schrodinger software, after which the partial charges were assigned, followed by Ligprep.^52^ For the **PQQ16P** SuMD simulation, the ligand parameters were achieved from the Paramchem service (CGenFF).^46^ Since the ligands’ behavior did not show penalties, we decided to use these parameters for SuMD simulation.

### 2.3 MD System Setup

After NorA system preparation, the transmembrane protein in apo form (without the Fab) was embedded in a POPC lipid bilayer, according to the suggested orientation reported in the OPM database. Initial POPC atoms were placed through the VMD membrane builder plugin,^53^ and lipids within 0.6 Å from amino acid atoms were removed. The membrane used in all of the simulations has a dimension of 120 × 120 Å. The system was solvated with TIP3P water using the program Solvate 1.0^54^ and neutralized by Na^+^/Cl^–^ counterions to a final concentration of 0.154 M. The system was then equilibrated through three main steps of molecular dynamics to equilibrate them. In the first stage, after 1500 steps of minimization to allow the system to reduce the clashes between proteins and lipids, 5 ns of MD simulation (2 500 000 steps) was performed in the NPT ensemble, restraining the ligand, protein atoms, and phosphorus of the phospholipid by a positional constraint of 1 kcal mol^–1^ Å^–2^. The temperature was maintained at 310 K using a Langevin thermostat with a low damping constant of 1 ps^–1^. The pressure was maintained at 1 atm using a Berendsen barostat; bond lengths involving hydrogen atoms were constrained using the M-SHAKE algorithm with an integration time step of 2 fs. In the second stage, applying the restraints only to the protein and to the ligand and keeping the conditions of constant pressure and temperature (NPT), the temperature was set at 310 K and the pressure at 1 atm, and 10 ns of MD was performed. Then, the last equilibration step included 20 ns of MD simulation, and the only restraints left were on the α carbon of amino acids and on the ligand.

### 2.4 MD Simulations

MD simulations of 100 ns of the system (NorA was in apo form) were performed using the ACEMD (vers. 2018.11.26) engine with a time step of 2 fs. The MD trajectory was staged at 5000 frames. The protein RMSD and RMSF were computed on the protein Cα using the VMD trajectory tool.^53^

### 2.5 SuMD System Setup

The prepared NorA protein was complexed with the **PQQ16P** derivative. The position of the ligand was manually assigned. To avoid protein–ligand long-range interactions, in the starting geometry, **PQQ16P** was positioned 61 Å from the NorA efflux pump atoms. The system for the simulation has been prepared with the same protocol described in section 2.3.

### 2.6 Supervised Molecular Dynamics (SuMD)

Each SuMD simulation is composed of a number of consecutive short unbiased MD simulations (600 ps, editable by the user) in which a supervision strategy, based on a tabu-search-like strategy, is applied at the end of each simulation (Table S1 in the Supporting Information). The supervised variable is the distance between the ligand and protein binding site center of mass that is maintained until the protein–ligand distance reaches a preset threshold value (5 Å in this case study). Then, the simulation proceeds as a conventional unbiased MD simulation. For a more detailed description of the SuMD analyzer, Salmaso et al.^42^ provide all of the necessary information. The SuMD code is available at this link (https://github.com/molecularmodelingsection/SuMD)

### 2.7 Analysis of SuMD Trajectories

All of the trajectories generated by SuMD were analyzed using an in-house script written in tcl and Python that makes use of MDtraj^55^ and ProDy^56^ modules. Most of the analyses are implemented in a Python tool available at this link (https://github.com/molecularmodelingsection/SuMD-analyzer). The analyses were then performed on the whole trajectories. In brief, the single SuMD step trajectories were stridden, by a user-defined value (here 20), superposed on the first frame Cα carbon atoms of the target protein, wrapped, and merged. The in-house script computed several steps of the SuMD simulation. It analyzes the geometry, such as the distance between the ligand and the binding site center of mass, and the protein RMSD, the ligand–target interaction energy estimation during the recognition process plotted on the Interaction Energy Landscape plots. For a more detailed description of the SuMD analyzer, Salmaso et al.^42^ provide all of the necessary information. This analysis also calculates all of the established interactions between the protein and the ligand.

The clustering analysis was performed using the density-based clustering algorithm DBSCAN,^57^ setting the RMSD threshold to 1.50 Å and the minimum number of protein conformations that could generate the cluster to 10 for NorA-**PQQ16P** system. The advantage of this algorithm is that it does not require an *a priori* definition of the number of clusters. Figures were generated by using Pymol.

### 2.8 MD Studies on Replica 5

Explicit solvent MD simulations were performed by using the Desmond package v6.6 (Schrödinger Release 2021–2)^31^ to investigate the stability of the SuMD binding mode generated in replica 5. The OPLS3e force field was selected, and the systems were solvated in an orthorhombic box (size = 20.0 × 20.0 × 20.0) exploiting the TIP3P water molecule mode. Na^+^ and Cl^–^ counterions at a concentration of 0.15 M were added to neutralize the system charge by means of the System Builder tool. The systems were relaxed before the simulation using the default protocol implemented in Desmond. The system minimization was followed by a production step of 1000 ns, which was simulated by the NTP ensemble at 310 K and 1 atm. MD simulation results were analyzed through the simulation interaction diagram (SID) tool provided by Schrödinger.

### 2.9 Molecular Docking Simulations

Docking studies were performed using Schrödinger’s Glide software.^58,59^ The NorA–**PQQ16P** complex conformation with the lowest energy was extracted from SuMD replica 5 (frame 308) used as a starting protein conformation for docking studies. Specifically, the protein was submitted to Schrodinger Protein Preparation Wizard.^50^ Then the H-bond network of the complex was optimized using PROPKA for the assignment of the residue protonation states (pH = 7.0), and the complex was submitted to a restrained minimization (OPLS3 force field) which was stopped when the RMSD of heavy atoms reached 0.30 Å.

Next, the position of **PQQ16P** in the conformation was used as a reference to center the grid. The docking space was defined as a cubic box (28 Å outerbox), with an inner cubic box (14 Å) defining the region where the center of mass (CM) of the ligand had to be located. The protein CM for replica 5 had coordinates of −0.16, −2.69, and 1.62 to the *x*-, *y*-, and *z*-axis, respectively. After grid preparation with the “receptor grid generation” tool, docking experiments were performed using the Glide XP (Extra Precision) protocol by generating one pose for each ligand. The generated poses were submitted to the MMGBSA minimization step using the “Refine Protein-Ligand Complex” tool in Prime (default settings—residues at 5 Å within the ligand considered as flexible).

The obtained ligand poses were finally clustered by computing the structural interaction fingerprint (SIFt)^60^ using the interaction fingerprint tool of Maestro. In particular, a SIFt was generated for each ligand protein complex generated by the MMGBSA minimization step, taking into account any contact or polar/nonpolar interaction that the ligand established with the protein backbone or side chains. The calculation of the Tanimoto similarity for the produced SIFts allowed their clusterization by applying the average linkage method as a distance metric. The best number of clusters was determined by considering the Kelley penalty.

## 3 Results and Discussion

### 3.1 Evaluation of Protein Stability Through Classical MD

**Figure 2:**
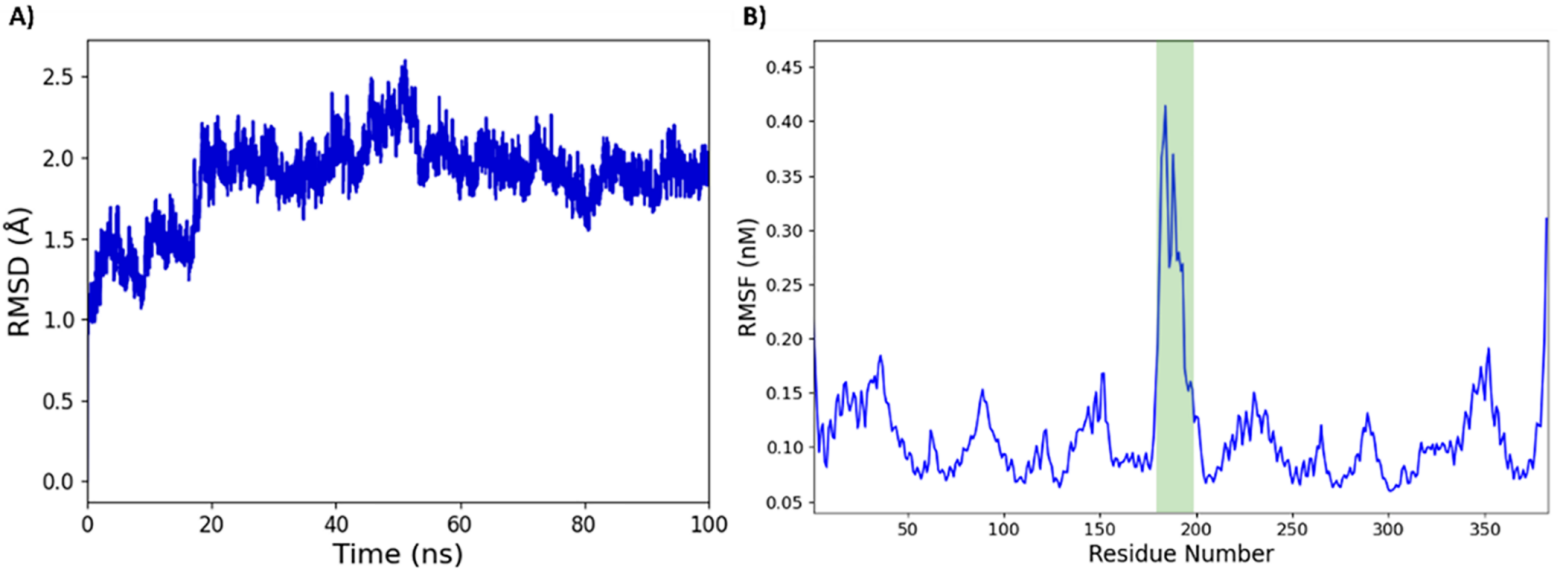
Assessment of the NorA efflux pump cryo-EM structure in a POPC bilayer. (A) Ligand root-mean-square deviation (RMSD) plot computed for the MD simulation trajectory. (B) Residues’ root-mean-square fluctuations (RMSF) during the MD simulation time of the NorA protein. The green area highlights the RMSF values of the flexible cytoplasmatic loop.

As a first, we investigated the stability of the NorA C_out_ conformation as derived by the 7LO8 structure. After the protein preparation step, the Fab36 portion was removed from the protein model, and the generated apo structure of the NorA efflux pump was embedded in a 1-palmitoyl-2-oleyl-glycerol-3-phospho-choline (POPC) bilayer. The protein was then subjected to 100 ns MD simulation. As shown in Figure 2A, the RMSD value of Cα atoms showed reasonable stability of the NorA structure, even in the absence of the Fab fragment. Specifically, only a slight perturbation was observed after the first 20 ns, followed by an overall stability achieved by the protein with its conformation remaining reasonably constant during the whole dynamic simulation time. Additionally, the most significant residue fluctuations occurred at the level of the C-term and N-term domains, as expected, as well as in the loop connecting helices 6 and 7 (residues 184–197 located in the cytoplasmatic portion of the protein, green area in Figure 2B). Hence, the fluctuation of this loop would not affect the ligand binding events sampled from the extracellular side of the protein. This finding demonstrated the overall protein stability, especially in the extracellular side of the structure, supporting its use in subsequent SuMD studies.

### 3.2 Inhibitor Binding Simulations using SuMD

Once the protein stability was assessed, SuMD simulations were applied to investigate the ligand binding pathway of the in-house EPI **PQQ16P**.

**PQQ16P** was selected for SuMD studies as the representative compound of a set of 38 in-house small molecules (compounds **1**–**37** and **PQQ16P**, hereafter called NorA active set) showing a strong NorA efflux pump inhibition activity (*i.e*., EtBr efflux inhibition ≥ 90% at a concentration equal to or lower than 50 μM). The compounds composing the NorA active set shared a very similar structural organization composed by (i) a positively charged group, (ii) a bicyclic, quinoline-like aromatic core (*i.e*., quinoline, quinazoline, or benzimidazole core), and (iii) a 2-phenylpropoxy moiety.

The SuMD method requires determination of the binding site to be reached by the ligand. Following the analysis of the available NorA structural data, the binding site was defined by the residues involved in the Fab36-NorA interaction and outlined as being important for the inhibitory activity of the antibody peptide. Specifically, Asn137, Phe140, Phe303, Asp307, Arg310, Glu222, and Thr223 were set to describe the site explorable by the inhibitor. Notably, Glu222 and Asp307 were reported as two key residues crucial for the binding of the Fab.^19^

**PQQ16P** was initially placed 61 Å away from the defined binding site, and then different replicas were performed to simulate the binding event of the ligand to the NorA protein. In total, seven simulations were produced. Since we were particularly interested in those recognition events reaching the deepest part of the NorA channel in its outward conformation, we classified the replicas as productive and nonproductive according to the distance between the CM of the ligand and that of the binding site (CM distance, Table 1). In two of the seven replicas (*i.e.*, 4 and 6), the ligand failed to enter the NorA channel, as indicated by the observed CM distance that remained above 20 Å. In replicas 1, 3, and 7, the ligand approached but did not reach the site. Indeed, in the performed simulation, the SuMD supervision stopped at a CM distance less than 5 Å (see Methods), which was the minimum distance below which a ligand can be considered to occupy the defined site. The CM-distance values obtained for the three mentioned replicas (*i.e.*, CM distance > 5 Å) indicated that the ligand was likely exploring a metastable state that prevented further ligand proximity to the defined binding site. Finally, two productive replicas were obtained, *i.e*., replicas 2 and 5. In these replicas, the ligand followed two different entrance pathways, and the most relevant explored steps are resumed in the next paragraphs. The 2:7 ratio obtained for productive over nonproductive replica was in line with our expectation based on the observation achieved for other transmembrane proteins. Of note, we previously noted that the main aspect affecting this ratio is not the affinity between the small molecule and the binding site^38,44,61^ but the complexity of the recognition event at the molecular level (*e.g.,* target and ligand flexibility, peculiar molecular mechanism).^62^

**Table 1:** SuMD Replicas Result Summary^a^

system	replica	outcome	time (ns)	CM distance (Å)
NorA-**PQQ16P**	1	nonproductive	51	7.66
	2	productive	51	4.43
	3	nonproductive	40	6.2
	4	nonproductive	26.4	22.43
	5	productive	26	2.95
	6	nonproductive	51	23.51
	7	nonproductive	51	7.33

a The final simulation time and the lowest CM distance (the distance between the mass centres of the ligand and of the binding site) reached by the system is reported for each replica.

#### 3.2.1 NorA-PQQ16P Recognition Pathway

**Figure 3:**
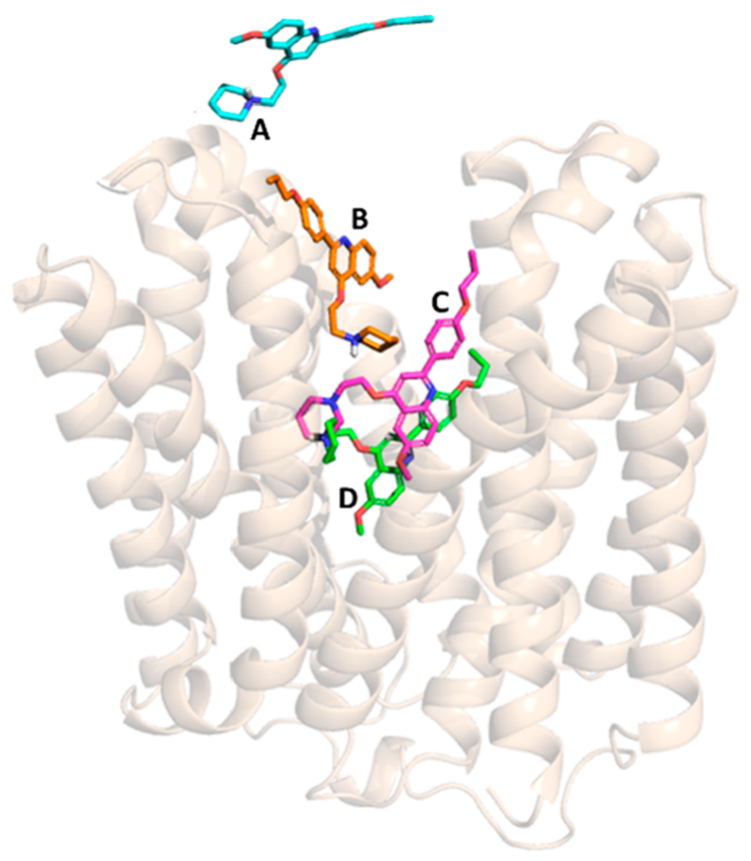
NorA-**PQQ16P** recognition pathway during SuMD replica 2.

**Figure 4:**
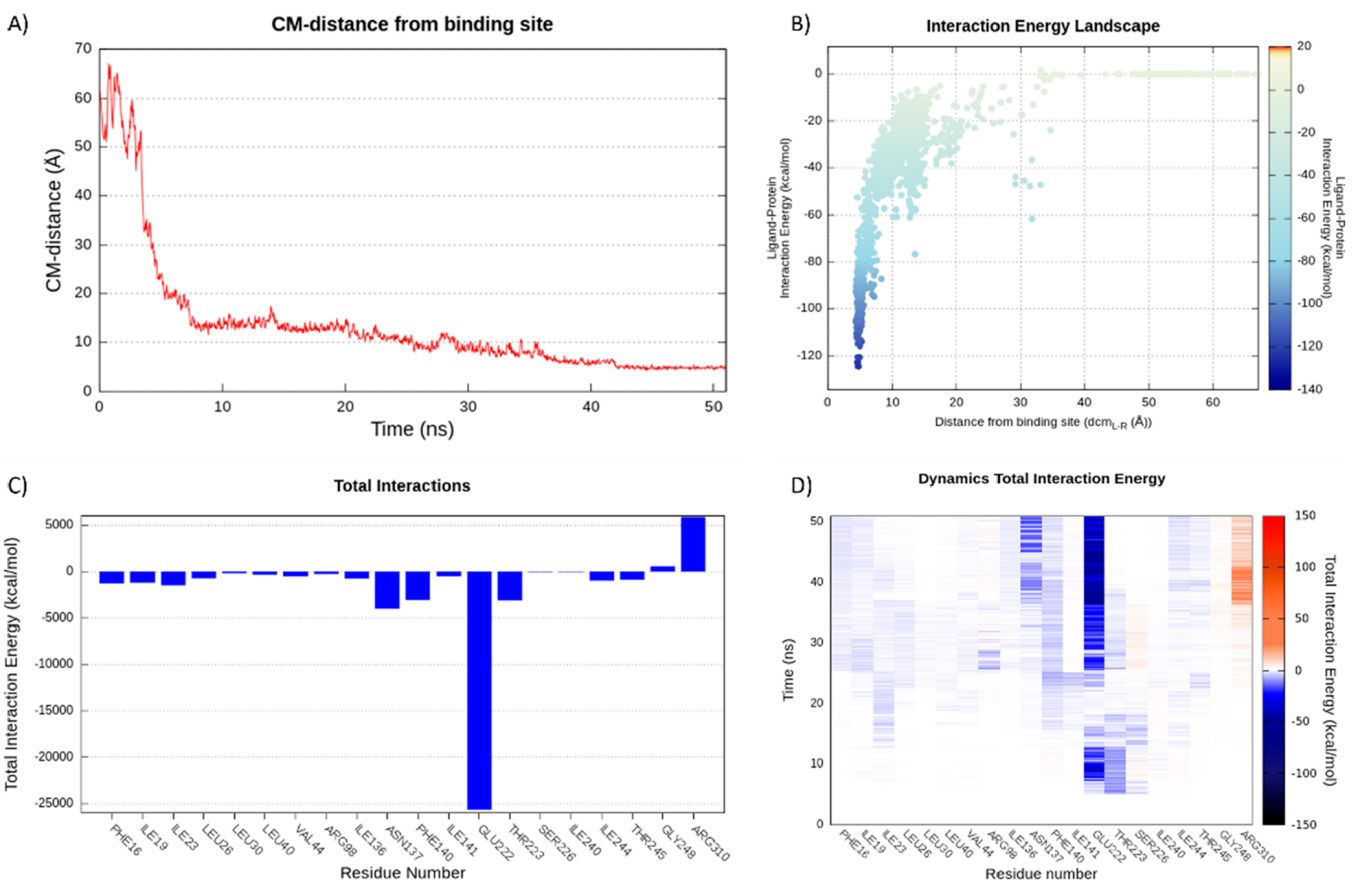
SuMD NorA-**PQQ16P** recognition pathway analysis in replica 2. (A) CM distance between the ligand and the binding site, where CM-distance indicates the distance between the mass centers of the ligand and of the binding site. (B) Interaction energy landscape. (C) Cumulative total interaction energy plot: the electrostatic and van der Waals contributions to the potential energy for each frame were summed to obtain the cumulative total interaction energy. (D) Dynamics total interaction energy (electrical and van der Waals contributions to potential energy) for each ligand-interacting residue.

The first intermolecular interaction between the protein and the ligand occurred after 4 ns of productive trajectory, involving the negative side chain of Asp352 and the piperidine moiety of **PQQ16P**, respectively (position A in Figure 3). Then, the distance between the ligand and protein CMs rapidly decreased to about 12 Å (Figure 4 plot A). In the new position, the ligand started to establish interactions with the key residue Glu222, located in the transmembrane helix TMH7. This binding mode was also stabilized by hydrophobic interactions driven by Ile244, Ile240, and Pro237 (position B in Figure 3). At about 25 ns of the simulation time, **PQQ16P** reached a metastable state (position C in Figure 3) that was stabilized by an extensive and robust network of intermolecular interactions (Figure 4, plot D).

Specifically, (i) the positive charge of the protonated amine interacted with the negative carboxylic group of the Glu222, (ii) Arg98 established H bonds with the oxygen atom of the propoxy group, and (iii) the quinoline scaffold was stabilized by π–π interaction engaged with Phe140. Additionally, several hydrophobic contacts were established between residues Phe16, Ile19, Ile23, Leu26, and Leu30 in the TMH1–TMH4 region and apolar parts of the molecule. The stability of the ligand in this first site was also confirmed by the low energy conformation state that it achieved (Figure 4, plot B).

The **PQQ16P** EPI was hosted in this region until 40 ns of the productive trajectory, after which it moved to a more stable binding site. In the new ligand binding pose (position D in Figure 3), the positive charge of the protonated amine still strongly interacted with the negative carboxylic group of the Glu222. Additionally, the oxygen atom of the methoxy group gained interactions with Asn137. Furthermore, hydrophobic contributions by Phe140 and Ile19 were also observed. Of note, all of these key interactions were retained until the end of the simulation (Figure 4 plot D). The minimum observed value of the CM distance was 4.3 Å, and **PQQ16P** remained in this state until the end of the SuMD simulation occurred at 51 ns.

#### 3.2.2 NorA-PQQ16P Recognition Pathway: Replica 5

**Figure 5:**
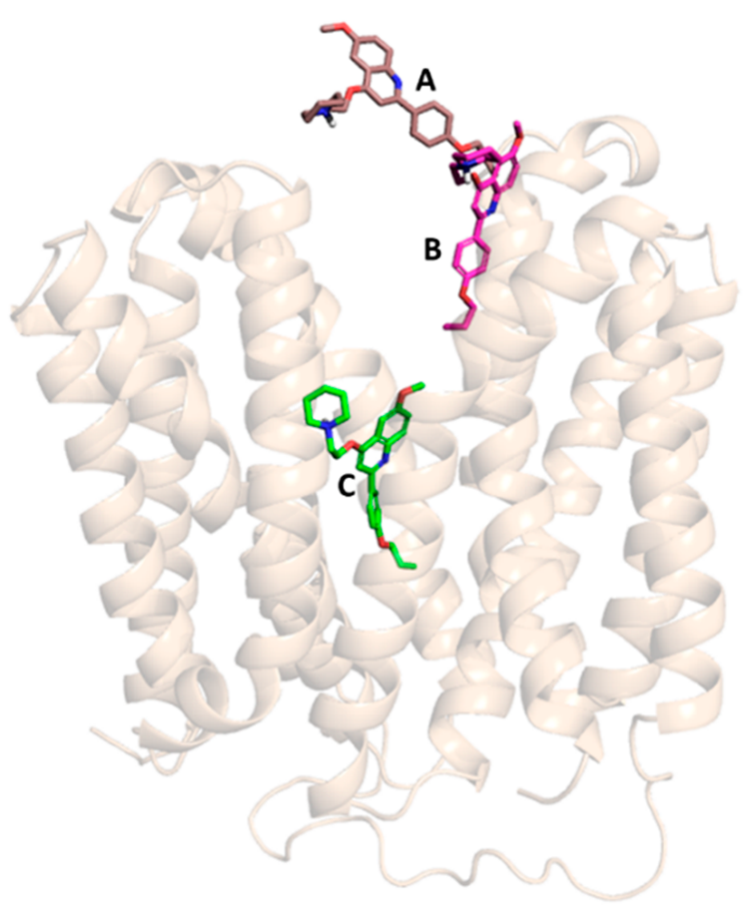
NorA-**PQQ16P** recognition pathway during SuMD replica 5.

**Figure 6:**
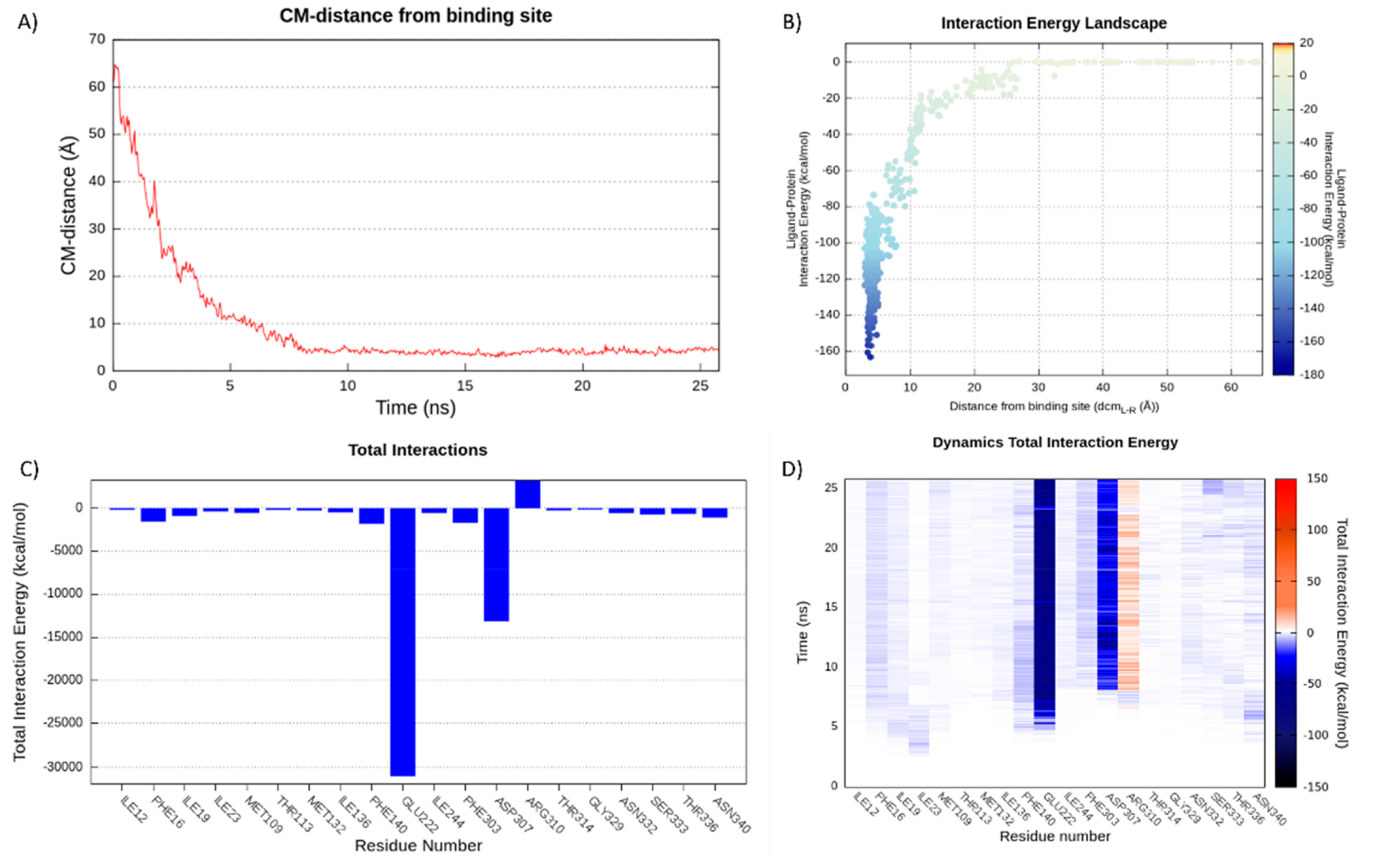
SuMD NorA-**PQQ16P** recognition pathway analysis, replica 5. (A) CM distance between the ligand and the binding site, where CM-distance indicates the distance between the mass centers of the ligand and of the binding site. (B) Cumulative total interaction energy plot: the electrostatic and van der Waals contributions to the potential energy for each frame were summed to obtain the cumulative total interaction energy. (D) Dynamics total interaction energy (electrostatic and van der Waals contributions to potential energy) for each ligand-interacting residue.

The first contact between the **PQQ16P** and the protein occurred after 2 ns of a productive trajectory. The recruitment of the ligand was driven by hydrophobic residues such as Leu40, Leu30, and Pro27 in TMH4 and TMH5, which hydrophobically interacted with the propoxy moiety of the EPI. The ligand had the piperidine and methoxy group facing outward from the pump, while the propoxy moiety was placed toward the inner part of the pump (position A in Figure 5). This represented the first recognition site in the vestibular region where the EPI was retained a few nanoseconds before rapidly moving inward to a deeper region. Then, the distance between the centers of mass of **PQQ16P** and NorA rapidly decreased to about 10 Å (plot A in Figure 6). Additionally, this quick distance reduction was in line with the observed energy behavior (plot B in Figure 6). As a matter of fact, the energy profile swiftly dropped from −20 kcal/mol (CM distance ≈ 20 Å) to −160 kcal/mol, when the CM distance reached around 5 Å (plot B in Figure 6).

After about 8 ns of simulation time, our EPI assumed a binding mode that was retained until the end of the simulation at 25 ns (position C in Figure 5). Indeed, as evident from the interaction energy landscape plot (plot B in Figure 6), the ligand achieved a stable conformation with an associated energy of −163.22 kcal/mol at about 6 ns. In this conformation, **PQQ16P** strongly interacted with both Glu222 and Asp307 via the protonated piperidine, while the quinoline nucleus was involved in an aromatic interaction with Phe140 until the end of the simulation (plot D in Figure 6). Relevant hydrophobic contributions were provided by Ile19, Ile136, Phe140, Phe16, Thr336, Thr113, and Thr314. It is worth noting that the minimum value of the observed CM distance was 2.95 Å.

#### 3.2.3 Identification of the Best Target Conformation for SBDD

SuMD simulations provided information on different protein states and regions explored by the quinoline **PQQ16P** when interacting with the NorA efflux pump. A comparative analysis between the two productive replicas underlined the presence of some key residues conserved in ligand binding. The residues most frequently contacted by **PQQ16P** in the two productive replicas were Glu222 and Phe140.

Next, the **PQQ16P** pathways during the SuMD productive trajectories (*i.e*., replica 2 and 5) were sampled by performing a clustering analysis using DBSCAN.^57^ The cluster analysis on the pathway explored by the investigated EPI allowed the identification of the most relevant states explored by the ligand during the simulated binding events. The DBSCAN algorithm identified clusters of ligand positions during each of the two SuMD replica simulations, thus highlighting which protein regions were mostly explored by the EPI. Generally, the cluster size is a result of the stability of the state; therefore, the identification of the largest cluster indicates the presence of stable or metastable sites. DBSCAN analysis of replica 2 revealed that cluster 11 was the most populated (Table 2). This cluster represented the last state explored by **PQQ16P** and corresponded to the last and most stable position that the ligand was able to explore.

**Table 2:** Summary of DBSCAN Clustering Performed on the Two Replicas^a^

	replica 2 (51 ns)		replica 5 (26 ns)	
cluster	#frames (% occ)	CM distance (Å)	#frames (% occ)	CM distance (Å)
0	18 (1.4)	13.72	407 (63)	4.13
1	11 (0.86)	12.67		
2	15 (1.17)	13.49		
3	14 (1.09)	12.37		
4	26 (2)	12.58		
5	36 (2.82)	9.83		
6	145 (11.4)	7.12		
7	9 (0.7)	7.40		
8	11 (0.9)	6.10		
9	28 (2.2)	5.77		
10	14 (1.1)	6.50		
11	244 (19.1)	4.76		

a For each cluster is reported the number of frames (#frames), the occurrence during the SuMD simulation expressed as a percentage (% occ), and the lowest CM distance (the distance between the mass centres of the ligand and of the binding site) in the cluster.

In this regard, the energy associated with each ligand–protein complex in cluster 11 (replica 2) ranged from −41.75 to −124.47 kcal/mol (Figure S1 in the Supporting Information).

It should also be noted that the final state reached by the ligand was preceded by a slightly less populated state (cluster 6 in Table 2). Even though this state was less relevant in terms of occurrence, it resembled a metastable site, therefore a state that appeared to have a significant impact on the ligand-protein recognition pathway.

However, the most relevant complex emerged from replica 5. According to the results of the analysis, **PQQ16P** seemed to have an immediate recognition pathway. Of note, replica 5 DBSCAN analysis showed a unique final state that persisted for most of the SuMD simulation time. When compared to the final position reached by **PQQ16P** in the other replica (*i.e*., cluster 11 in replica 2), the unique cluster generated for replica 5 was the most populated state occurring for 63% of the trajectory. In general, all of the protein–ligand conformations belonging to this cluster had an associated energy ranging from −73.44 to −163.22 kcal/mol (Figure S1 in Supporting Information).

**Figure 7:**
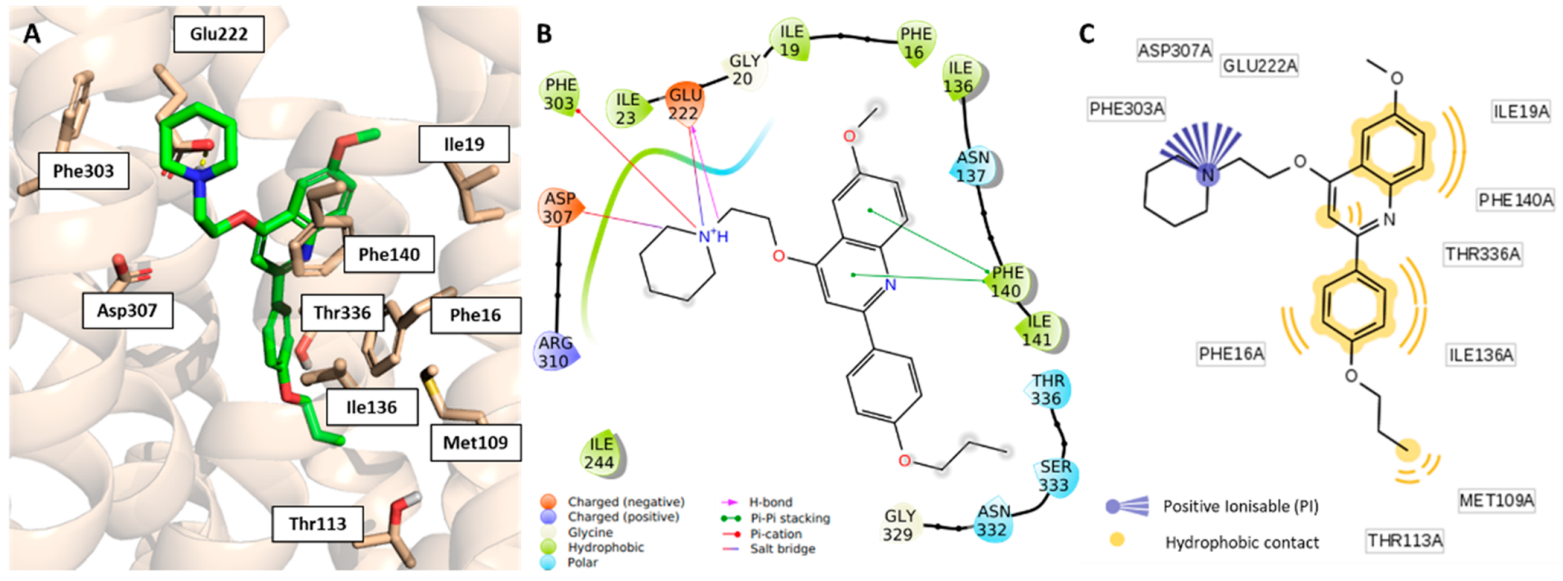
Representation of the **PQQ16P** SuMD binding pose into the NorA efflux pump. (A) 3D structure of the lowest ligand energy conformation in replica 5. 2D schematic representation of key intermolecular interactions as suggested by the Ligand Interaction tool of Maestro (B) and LigandScout (C).

In the lowest energy conformation (*i.e*., that one at −163.22 kcal/mol; Figure 7), the protonated amine of **PQQ16P** was involved in a salt bridge with Glu222 and Asp307, and in a π-cation interaction with Phe303. Additionally, the two rings of the quinoline scaffold made both π–π and hydrophobic interactions with Phe140.

### 3.3 *In Silico* Characterization of in-House EPIs-NorA Interactions

The information gathered from the binding events sampled in the SuMD calculations led to the identification of the final binding observed in replica 5 as the most relevant state explored by **PQQ16P** within the NorA channel since it (i) was the easiest to reach, (ii) was explored for the longest period, (iii) had the lowest associated energy, and (iv) achieved the closest distance to the predefined site. Additionally, in order to probe the residence of **PQQ16P** in the binding site, we carried out an extended 1 μs MD simulation of replica 5. The results of the analysis strongly indicated that the SuMD pose exhibited an overall stable behavior characterized by a conserved position during the whole simulation (Figure S2 in the Supporting Information).

As already mentioned, **PQQ16P** was selected for SuMD studies as a representative compound of the NorA active set. The comparable biological effect observed for the most potent EPIs should in principle depend on conserved intermolecular interactions established with their biological target, *i.e*., NorA. Although the key ligand chemical features had already been suggested through a ligand-based approach (*i.e*., the 3D-pharmacophore model),^63^ here we aimed at exploring the new structural information to propose a shared pattern of ligand–protein interaction for the most active EPIs. To address this task, the NorA active set and a newly defined NorA inactive set (*i.e*., EtBr efflux inhibition ≤ 30% at a concentration equal to or lower than 50 μM, compounds **38**–**102**) were submitted to molecular docking experiments against the protein conformation extracted from the NorA-**PQQ16P** complex generated in replica 5. Specifically, the *in silico* protocol consisted of Glide XP followed by Prime minimization and MMGBSA rescoring.

The docking results were evaluated based on various criteria, including the protocol capacity to (i) reproduce the SuMD binding mode of **PQQ16P** and (ii) predict a consistent binding mode.^60^ The generated SIFs were finally clusterized based on their similarity, resulting in 15 different binding pose clusters. Importantly, the binding mode identified for **PQQ16P** (binding pose cluster 12 in Table S2) was also produced for 31 out of the 38 EPIs in the NorA active set (Table S2 in Supporting Information). Of note, by focusing on the compounds with the higher EPI activity (*i.e*., the 50 compounds with an EtBr efflux inhibition ≥ 95%), all but one produced a predicted protein-bound conformation comparable to that generated for **PQQ16P**.

The in-depth analysis of the binding poses and ligand–protein interactions generated for this specific subset of compounds is summarized in Table 3.

**Table 3:**
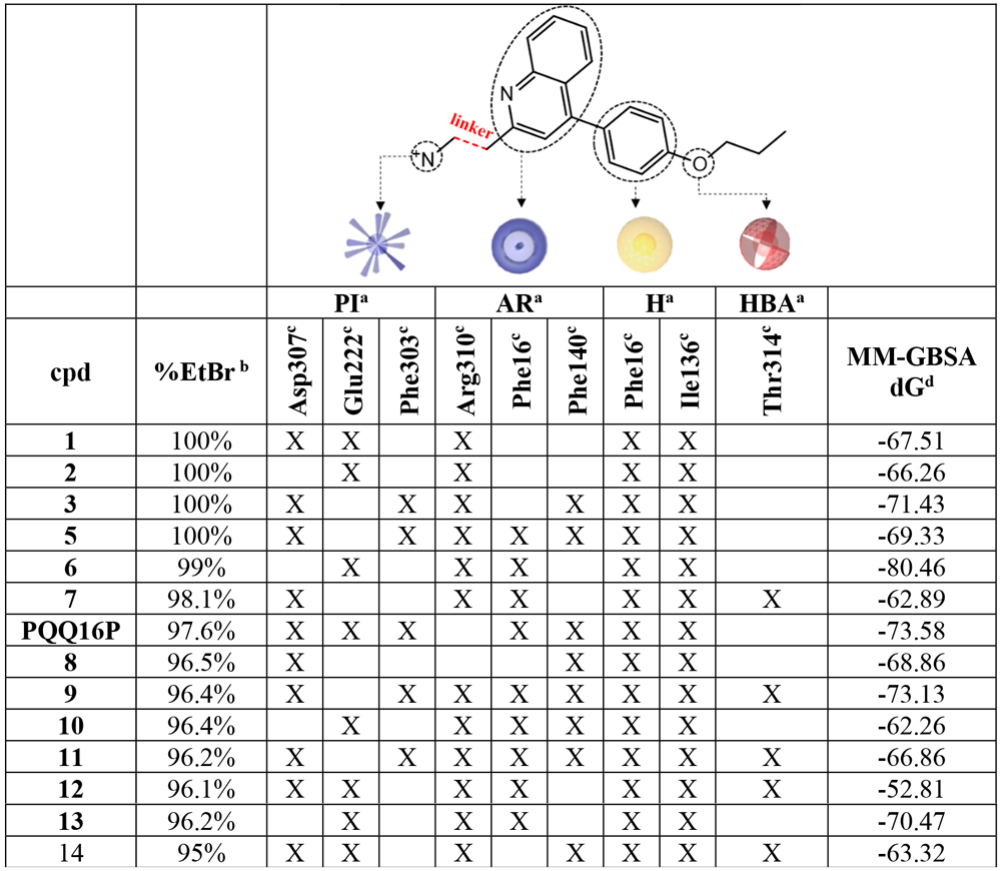
Ligand Chemical Features and Protein Residues Involved in the EPI–NorA Interaction for the in House Potent EPIs (EtBr Efflux Inhibition ≥ 95%) Belonging to Binding Pose Cluster 12

a PI, positive ionizable; AR, aromatic ring; H, hydrophobic region; HBA, H-bond acceptor.

b %EtBr, percentage of EtBr efflux inhibition.

c The protein–ligand interactions were retrieved from the Ligand Interaction tool of Maestro and LigandScout.

d MM-GBSA dG: MMGBSA Δ*G* of binding (kcal/mol).

At first, the presence of a positively charged moiety was confirmed to be essential, as it strongly interacted with residues Glu222, Phe303, and Asp307. These three residues formed an electron-rich area that could establish strong polar contacts with the ligand.

The bicyclic aromatic core of the NorA EPIs was placed between Phe16, Phe140, and Arg310 and performed hydrophobic/stacking interactions with these residues. Additionally, all compounds showed a hydrophobic phenyl moiety located in the proximity of Phe16 and Ile136. Finally, in some EPIs, an acceptor group was able to establish a H-bond interaction with the Thr314 side chain.

**Figure 8:**
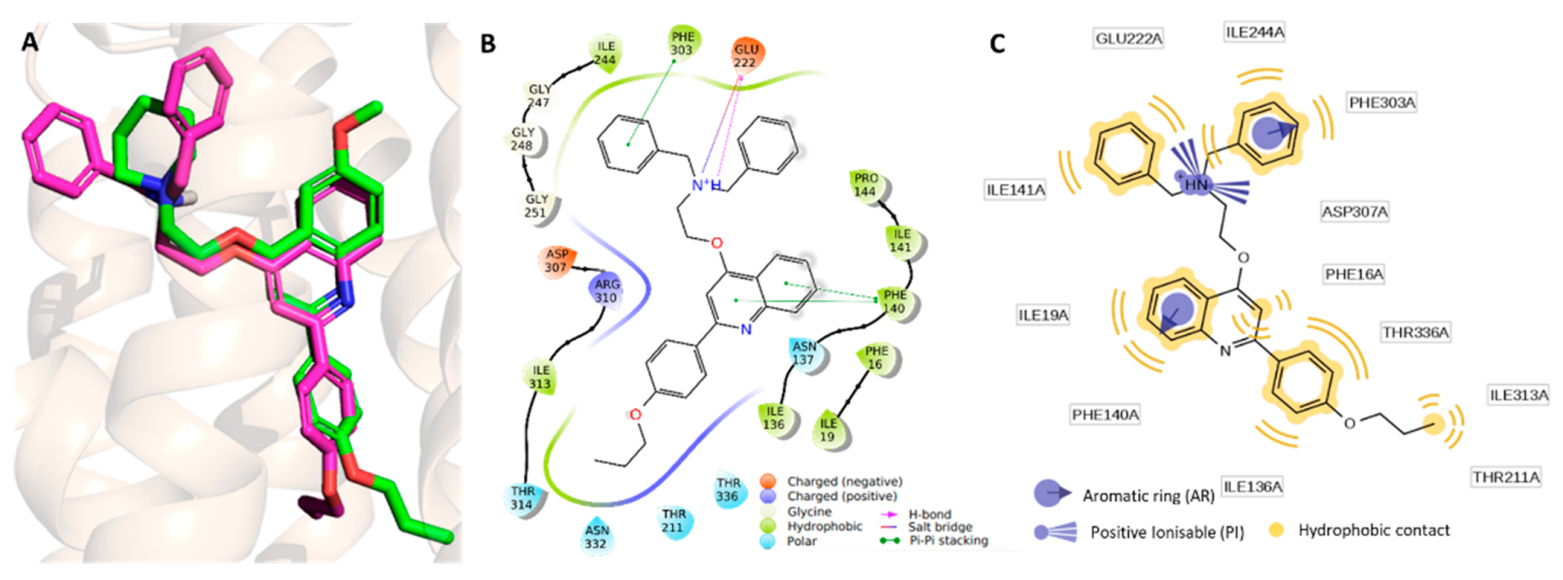
Representation of **39**’s binding mode into the NorA efflux pump. (A) Superimposition between the PQQ16P SuMD-derived pose (green) and Glide XP (magenta) predicted binding mode of **39**. 2D schematic representation of key intermolecular interactions as suggested by the Ligand Interaction tool of Maestro (B) and LigandScout (C).

Regarding the NorA inactive set, only compounds **38** and **39** (EtBr efflux inhibition = 0%; Table S2 in Supporting Information) were collected in the binding pose cluster 12, as for PQQ16P. However, while compound **38** did not possess any protonable group, compound **39** fitted all of the chemical requirements highlighted as important for the active compounds (Figure 8).

Nevertheless, an in-depth analysis of **39** using MoKa,^64^ a software able to accurately predict p*K*_a_ values and tautomerism in the aqueous medium with the respective species abundance, suggested that the calculated p*K*_a_ of the tertiary nitrogen atom was 7.05. Thus, under physiological conditions, the abundance of the charged form was lower than 50% (*i.e*., 40.8%), suggesting the lack of a “strong” positive ionizable group in this inactive compound.

**Figure 9:**
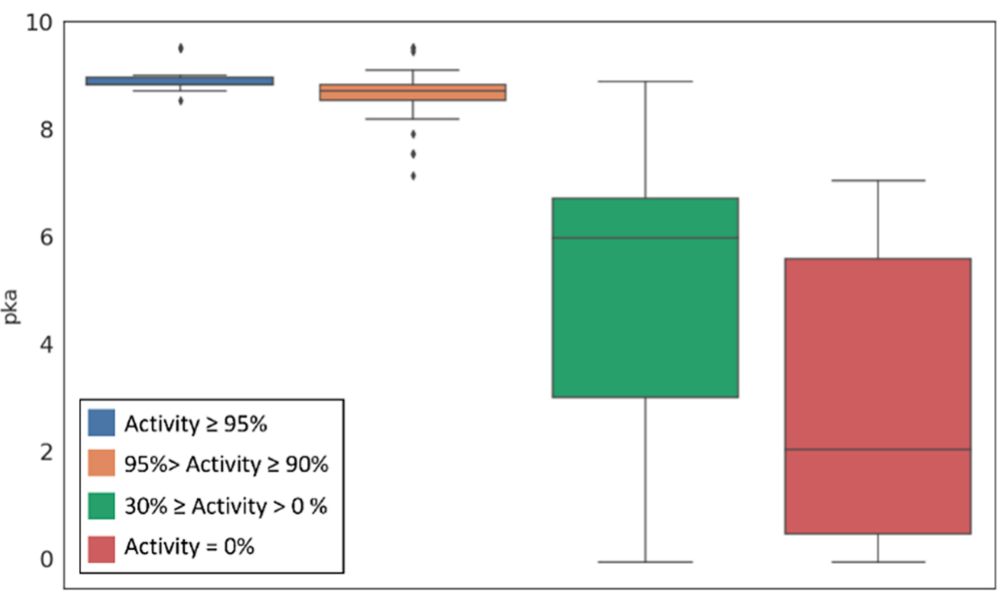
Box plots of the distribution of the p*K*_a_ values calculated on the most basic center for each compound composing the NorA active and inactive sets. The activity thresholds refer to the percentage of EtBr efflux inhibition at a concentration equal to or lower than 50 μM.

This evidence was consistent with the trend observed in the compounds composing the active and inactive sets. Indeed, moving from the potent EPIs to the inactive compounds, we observed a shift in the p*K*_a_ value of the most basic center predicted by MoKa (Figure 9). In particular, the highly active compounds were characterized by a basic center with a p*K*_a_ value greater than 8.5.

Overall, the results demonstrated the capacity of the docking protocol to identify for the active compounds a common interaction pattern with the NorA protein and to distinguish active from inactive molecules.

## 4 Conclusions

In this work, the SuMD methodology and docking experiments were combined to reveal for the first time the dynamic interaction pattern between EPIs and the NorA protein. Specifically, the use of SuMD allowed the reconstruction of a possible molecular recognition pathway of the in-house EPI **PQQ16P** by the efflux pump, from the initial unbound state to the final protein–ligand complex. Notably, **PQQ16P** rapidly reached a binding position associated with a very favorable energy after only about 8 ns, and this ligand pose was maintained for the remaining simulation time.

Subsequently, molecular docking experiments revealed that the binding mode identified by SUMD for **PQQ16P** was also shared by the most effective EPIs, as demonstrated by the fact that 13 of the remaining 14 highly active 2-phenylquinoline analogues (*i.e*. compounds with an EtBr efflux inhibition of 95%) displayed the same predicted inhibitor-bound conformation as that of NorA.

**Figure 10:**
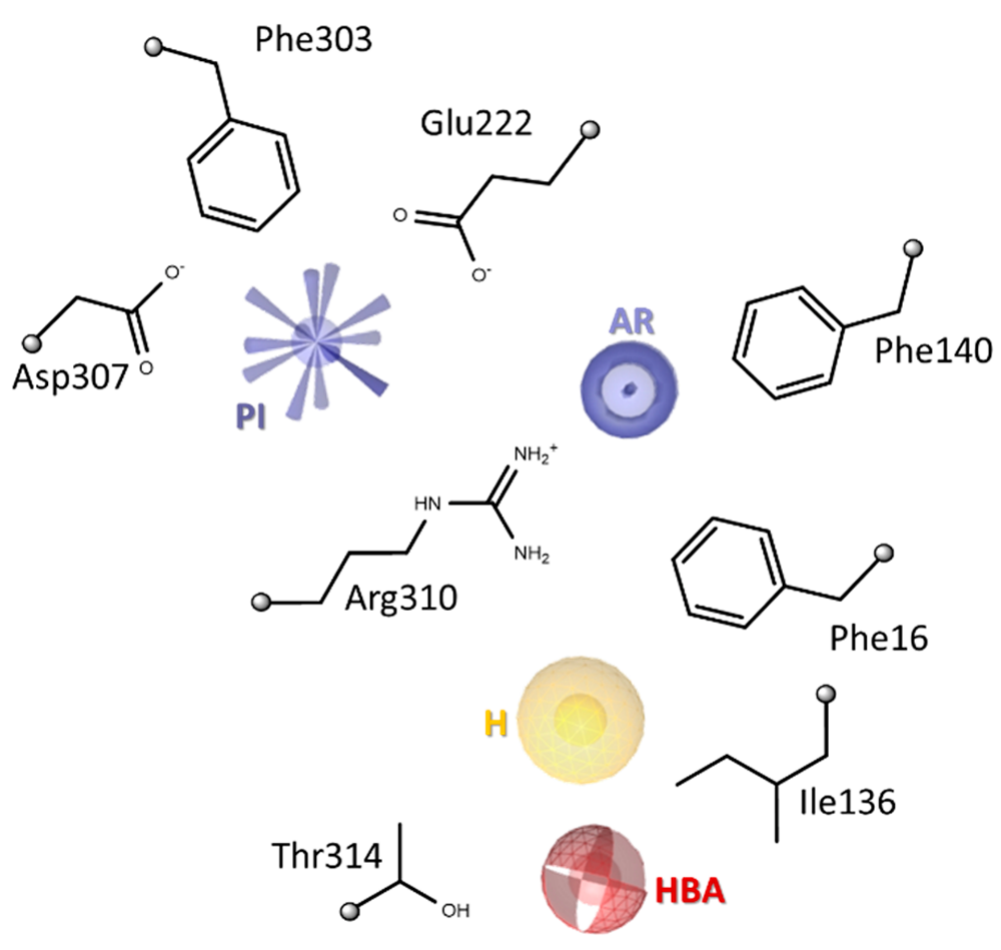
Schematic representation of the ligand chemical features and protein residues involved in the EPI-NorA interaction. PI, positive ionizable; AR, aromatic ring; H, hydrophobic region; HBA, H-bond acceptor.

The analysis of the computed ligand–protein interactions for this set of potent EPIs (Table 3) enabled us to draw some structure-based considerations about the key chemical features responsible for the strong inhibitory activity of these compounds (Figure 10).

First, a positive ionizable group (*i.e*., p*K*_a_ > 8.5; PI) emerged to be a crucial element during the ligand–protein recognition process due to the attractive driving forces established between this moiety and the negatively charged amino acids Glu222 and Asp307. Furthermore, the compound’s positively charged portion strongly contributed to the stability of the final ligand-bound state due to its interactions with Phe303, Glu222, and Asp307.

Moving to the aromatic core (AR) of the quinoline derivatives, this part made strong π-stacking contacts with Phe16, Phe140, and Arg310 residues, highlighting the significance of the bicyclic aromatic system as a central scaffold.

Concerning the 2-phenyl propoxy moiety, the hydrophobic aromatic region (H) formed well-conserved interactions with Phe16 and Ile136 in all of the investigated EPIs.

Notably, the chemical features uncovered as critical for ligand–protein recognition in this study are quite in agreement with the findings reported in our previous ligand-based work,^63^ where we described the development of common feature pharmacophore models (Figure S4) for potent NorA EPIs belonging to different chemical families. Indeed, the best 3D model contained four pharmacophore sites, consisting of one positive charge, one aromatic ring, one hydrophobic region, and one H-bond acceptor. Now, structure-based simulations and analyses have independently revealed that the first three of the previously identified elements—here named PI, AR, and H (Figure 10)—were crucial for NorA recognition and binding, thereby definitely confirming these chemical features as essential requirements for potent EPIs.

Regarding the H-bond acceptor identified during the ligand-based pharmacophore generation,^63^ this element has been found in the docking poses of only five of the 14 compounds, where the oxygen atom of the 2-phenyl propoxy moiety was able to establish H-bond interaction with the Thr314 side chain (Figure S5). As a result, it is difficult to establish the real significance of this chemical characteristic for which more in-depth investigations are required. Of note, it is well-known that ligand- and structure-based methods can be combined to enhance the reliability and efficiency of computer-aided drug discovery strategies,^65,66^ with several examples reported for different protein targets.^67,68^ In this context, the outcomes of our earlier and current investigations set the groundwork for the development of integrated virtual screening pipelines specifically designed for the NorA efflux pump.

In conclusion, this research work represents a step forward in deciphering the intricate mechanism governing NorA-EPI recognition and provides useful information and data to support the fight against AMR. Indeed, we release the 3D model structure of compound **PQQ16P** bound to NorA as obtained by the SuMD simulations and used in molecular docking experiments. Hence, computational researchers interested in searching for novel potential NorA EPIs from different chemical families could explore the proposed ligand binding region in structure-based virtual screening campaigns.

## Data Availability

Topology, parameter, and coordinates files as well as MD trajectories presented in the manuscript are available at 10.5281/zenodo.7798718.

## Supporting Information Available

Supplementary figures and tables; compound data sets composition with associated chemical structures as smiles, biological activity, MMGBSA dG Bind, and binding mode cluster (PDF)
SuMD-derived **PQQ16P**-NorA complex (PDB)

## Supplementary Material

ci3c00516_si_001.pdf

ci3c00516_si_002.pdb

## Abbreviations

AMR: antimicrobial resistance
CPX: ciprofloxacin
EtBr: ethidium bromide
EPI: efflux pump inhibitor
SuMD: supervised molecular dynamics
MD: molecular dynamics
CM: center of mass

## References

[ref1] Davies J.; Davies D. Origins and evolution of antibiotic resistance. Microbiol Mol. Biol. Rev. 2010, 74, 417–433. 10.1128/MMBR.00016-10. 20805405 PMC2937522

[ref2] Brown E. D.; Wright G. D. Antibacterial drug discovery in the resistance era. Nature 2016, 529, 336–343. 10.1038/nature17042. 26791724

[ref3] Murray C. J. L.; Ikuta K. S.; Sharara F.; Swetschinski L.; Robles Aguilar G.; Gray A.; Han C.; Bisignano C.; Rao P.; Wool E.; Johnson S. C.; Browne A. J.; Chipeta M. G.; Fell F.; Hackett S.; Haines-Woodhouse G.; Kashef Hamadani B. H; Kumaran E. A. P.; McManigal B.; Achalapong S.; Agarwal R.; Akech S.; Albertson S.; Amuasi J.; Andrews J.; Aravkin A.; Ashley E.; Babin F.-X.; Bailey F.; Baker S.; Basnyat B.; Bekker A.; Bender R.; Berkley J. A.; Bethou A.; Bielicki J.; Boonkasidecha S.; Bukosia J.; Carvalheiro C.; Castaneda-Orjuela C.; Chansamouth V.; Chaurasia S.; Chiurchiu S.; Chowdhury F.; Clotaire Donatien R.; Cook A. J.; Cooper B.; Cressey T. R.; Criollo-Mora E.; Cunningham M.; Darboe S.; Day N. P. J.; De Luca M.; Dokova K.; Dramowski A.; Dunachie S. J.; Duong Bich T.; Eckmanns T.; Eibach D.; Emami A.; Feasey N.; Fisher-Pearson N.; Forrest K.; Garcia C.; Garrett D.; Gastmeier P.; Giref A. Z.; Greer R. C.; Gupta V.; Haller S.; Haselbeck A.; Hay S. I.; Holm M.; Hopkins S.; Hsia Y.; Iregbu K. C.; Jacobs J.; Jarovsky D.; Javanmardi F.; Jenney A. W. J.; Khorana M.; Khusuwan S.; Kissoon N.; Kobeissi E.; Kostyanev T.; Krapp F.; Krumkamp R.; Kumar A.; Kyu H. H.; Lim C.; Lim K.; Limmathurotsakul D.; Loftus M. J.; Lunn M.; Ma J.; Manoharan A.; Marks F.; May J.; Mayxay M.; Mturi N.; Munera-Huertas T.; Musicha P.; Musila L. A; Mussi-Pinhata M. M.; Naidu R. N.; Nakamura T.; Nanavati R.; Nangia S.; Newton P.; Ngoun C.; Novotney A.; Nwakanma D.; Obiero C. W.; Ochoa T. J.; Olivas-Martinez A.; Olliaro P.; Ooko E.; Ortiz-Brizuela E.; Ounchanum P.; Pak G. D.; Paredes J. L.; Peleg A. Y.; Perrone C.; Phe T.; Phommasone K.; Plakkal N.; Ponce-de-Leon A.; Raad M.; Ramdin T.; Rattanavong S.; Riddell A.; Roberts T.; Robotham J. V.; Roca A.; Rosenthal V. D.; Rudd K. E; Russell N.; Sader H. S.; Saengchan W.; Schnall J.; Scott J. A. G.; Seekaew S.; Sharland M.; Shivamallappa M.; Sifuentes-Osornio J.; Simpson A. J.; Steenkeste N.; Stewardson A. J.; Stoeva T.; Tasak N.; Thaiprakong A.; Thwaites G.; Tigoi C.; Turner C.; Turner P.; van Doorn H. R.; Velaphi S.; Vongpradith A.; Vongsouvath M.; Vu H.; Walsh T.; Walson J. L.; Waner S.; Wangrangsimakul T.; Wannapinij P.; Wozniak T.; Young Sharma T. E. M. W.; Yu K. C.; Zheng P.; Sartorius B.; Lopez A. D.; Stergachis A.; Moore C.; Dolecek C.; Naghavi M. Global burden of bacterial antimicrobial resistance in 2019: a systematic analysis. Lancet 2022, 399, 629–655. 10.1016/S0140-6736(21)02724-0. 35065702 PMC8841637

[ref4] Blair J. M.; Webber M. A.; Baylay A. J.; Ogbolu D. O.; Piddock L. J. Molecular mechanisms of antibiotic resistance. Nat. Rev. Microbiol 2015, 13, 42–51. 10.1038/nrmicro3380. 25435309

[ref5] Darby E. M.; Trampari E.; Siasat P.; Gaya M. S.; Alav I.; Webber M. A.; Blair J. M. A. Molecular mechanisms of antibiotic resistance revisited. Nat. Rev. Microbiol 2023, 21, 280 10.1038/s41579-022-00820-y. 36411397

[ref6] Khan S. N.; Khan A. U. Breaking the Spell: Combating Multidrug Resistant ’Superbugs’. Front Microbiol 2016, 7, 174 10.3389/fmicb.2016.00174. 26925046 PMC4757689

[ref7] Magiorakos A. P.; Srinivasan A.; Carey R. B.; Carmeli Y.; Falagas M. E.; Giske C. G.; Harbarth S.; Hindler J. F.; Kahlmeter G.; Olsson-Liljequist B.; Paterson D. L.; Rice L. B.; Stelling J.; Struelens M. J.; Vatopoulos A.; Weber J. T.; Monnet D. L. Multidrug-resistant, extensively drug-resistant and pandrug-resistant bacteria: an international expert proposal for interim standard definitions for acquired resistance. Clin Microbiol Infect 2012, 18, 268–281. 10.1111/j.1469-0691.2011.03570.x. 21793988

[ref8] Qadri H.; Shah A. H.; Mir M. Novel Strategies to Combat the Emerging Drug Resistance in Human Pathogenic Microbes. Curr. Drug Targets 2021, 22, 1424–1436. 10.2174/1389450121666201228123212. 33371847

[ref9] Tegos G. P.; Haynes M.; Strouse J. J.; Khan M. M.; Bologa C. G.; Oprea T. I.; Sklar L. A. Microbial efflux pump inhibition: tactics and strategies. Curr. Pharm. Des 2011, 17, 1291–1302. 10.2174/138161211795703726. 21470111 PMC3717411

[ref10] Wright G. D. Resisting resistance: new chemical strategies for battling superbugs. Chem. Biol. 2000, 7, R127–132. 10.1016/S1074-5521(00)00126-5. 10873842

[ref11] Morel C.; Stermitz F. R.; Tegos G.; Lewis K. Isoflavones as potentiators of antibacterial activity. J. Agric. Food Chem. 2003, 51, 5677–5679. 10.1021/jf0302714. 12952418

[ref12] Fang G. Y.; Mu X. J.; Huang B. W.; Jiang Y. J. Monitoring Longitudinal Trends and Assessment of the Health Risk of Shigella flexneri Antimicrobial Resistance. Environ. Sci. Technol. 2023, 57, 4971–4983. 10.1021/acs.est.2c08766. 36929874

[ref13] Barbosa da Costa N.; Hebert M. P.; Fugere V.; Terrat Y.; Fussmann G. F.; Gonzalez A.; Shapiro B. J. A Glyphosate-Based Herbicide Cross-Selects for Antibiotic Resistance Genes in Bacterioplankton Communities. mSystems 2022, 7, e0148221 10.1128/msystems.01482-21. 35266795 PMC9040730

[ref14] Foster T. J. The Staphylococcus aureus ″superbug″. J. Clin Invest 2004, 114, 1693–1696. 10.1172/JCI23825. 15599392 PMC535074

[ref15] Ippolito G.; Leone S.; Lauria F. N.; Nicastri E.; Wenzel R. P. Methicillin-resistant Staphylococcus aureus: the superbug. Int. J. Infect Dis 2010, 14, S7–S11. 10.1016/j.ijid.2010.05.003. 20851011

[ref16] Tong S. Y.; Davis J. S.; Eichenberger E.; Holland T. L.; Fowler V. G. Jr. Staphylococcus aureus infections: epidemiology, pathophysiology, clinical manifestations, and management. Clin Microbiol Rev. 2015, 28, 603–661. 10.1128/CMR.00134-14. 26016486 PMC4451395

[ref17] Costa S. S.; Viveiros M.; Amaral L.; Couto I. Multidrug Efflux Pumps in Staphylococcus aureus: an Update. Open Microbiol J. 2013, 7, 59–71. 10.2174/1874285801307010059. 23569469 PMC3617543

[ref18] Neyfakh A. A.; Borsch C. M.; Kaatz G. W. Fluoroquinolone resistance protein NorA of Staphylococcus aureus is a multidrug efflux transporter. Antimicrob. Agents Chemother. 1993, 37, 128–129. 10.1128/AAC.37.1.128. 8431010 PMC187619

[ref19] Brawley D. N.; Sauer D. B.; Li J.; Zheng X.; Koide A.; Jedhe G. S.; Suwatthee T.; Song J.; Liu Z.; Arora P. S.; Koide S.; Torres V. J.; Wang D. N.; Traaseth N. J. Structural basis for inhibition of the drug efflux pump NorA from Staphylococcus aureus. Nat. Chem. Biol. 2022, 18, 706–712. 10.1038/s41589-022-00994-9. 35361990 PMC9246859

[ref20] Stavri M.; Piddock L. J.; Gibbons S. Bacterial efflux pump inhibitors from natural sources. J. Antimicrob. Chemother. 2007, 59, 1247–1260. 10.1093/jac/dkl460. 17145734

[ref21] Seukep A. J.; Kuete V.; Nahar L.; Sarker S. D.; Guo M. Plant-derived secondary metabolites as the main source of efflux pump inhibitors and methods for identification. J. Pharm. Anal 2020, 10, 277–290. 10.1016/j.jpha.2019.11.002. 32923005 PMC7474127

[ref22] Lamut A.; Peterlin Masic L.; Kikelj D.; Tomasic T. Efflux pump inhibitors of clinically relevant multidrug resistant bacteria. Med. Res. Rev. 2019, 39, 2460–2504. 10.1002/med.21591. 31004360

[ref23] Sabatini S.; Kaatz G. W.; Rossolini G. M.; Brandini D.; Fravolini A. From phenothiazine to 3-phenyl-1,4-benzothiazine derivatives as inhibitors of the Staphylococcus aureus NorA multidrug efflux pump. J. Med. Chem. 2008, 51, 4321–4330. 10.1021/jm701623q. 18578473

[ref24] Fournier Dit Chabert J.; Marquez B.; Neville L.; Joucla L.; Broussous S.; Bouhours P.; David E.; Pellet-Rostaing S.; Marquet B.; Moreau N.; Lemaire M. Synthesis and evaluation of new arylbenzo[b]thiophene and diarylthiophene derivatives as inhibitors of the NorA multidrug transporter of Staphylococcus aureus. Bioorg. Med. Chem. 2007, 15, 4482–4497. 10.1016/j.bmc.2007.04.023. 17498961

[ref25] Fontaine F.; Hequet A.; Voisin-Chiret A. S.; Bouillon A.; Lesnard A.; Cresteil T.; Jolivalt C.; Rault S. First identification of boronic species as novel potential inhibitors of the Staphylococcus aureus NorA efflux pump. J. Med. Chem. 2014, 57, 2536–2548. 10.1021/jm401808n. 24499135

[ref26] Buonerba F.; Lepri S.; Goracci L.; Schindler B. D.; Seo S. M.; Kaatz G. W.; Cruciani G. Improved Potency of Indole-Based NorA Efflux Pump Inhibitors: From Serendipity toward Rational Design and Development. J. Med. Chem. 2017, 60, 517–523. 10.1021/acs.jmedchem.6b01281. 27977195

[ref27] Kumar A.; Khan I. A.; Koul S.; Koul J. L.; Taneja S. C.; Ali I.; Ali F.; Sharma S.; Mirza Z. M.; Kumar M.; Sangwan P. L.; Gupta P.; Thota N.; Qazi G. N. Novel structural analogues of piperine as inhibitors of the NorA efflux pump of Staphylococcus aureus. J. Antimicrob. Chemother. 2008, 61, 1270–1276. 10.1093/jac/dkn088. 18334493

[ref28] Oliveira-Tintino C. D. M.; Muniz D. F.; Barbosa C.; Pereira R. L. S.; Begnini I. M.; Rebelo R. A.; Silva L. E. D.; Mireski S. L.; Nasato M. C.; Krautler M. I. L.; Pereira P. S.; Costa J.; Rodrigues F. F. G.; Teixeira A. M. R.; Ribeiro-Filho J.; Tintino S. R.; de Menezes I. R. A.; Coutinho H. D. M.; Silva T. G. D. The 1,8-naphthyridines sulfonamides are NorA efflux pump inhibitors. J. Glob Antimicrob Resist 2021, 24, 233–240. 10.1016/j.jgar.2020.11.027. 33385589

[ref29] Pieroni M.; Sabatini S.; Massari S.; Kaatz G. W.; Cecchetti V.; Tabarrini O. Searching for innovative quinolone-like scaffolds: synthesis and biological evaluation of 2,1-benzothiazine 2,2-dioxide derivatives. Medchemcomm 2012, 3, 1092–1097. 10.1039/c2md20101a.

[ref30] Felicetti T.; Cannalire R.; Burali M. S.; Massari S.; Manfroni G.; Barreca M. L.; Tabarrini O.; Schindler B. D.; Sabatini S.; Kaatz G. W.; Cecchetti V. Searching for Novel Inhibitors of the S. aureus NorA Efflux Pump: Synthesis and Biological Evaluation of the 3-Phenyl-1,4-benzothiazine Analogues. ChemMedChem. 2017, 12, 1293–1302. 10.1002/cmdc.201700286. 28598572

[ref31] Felicetti T.; Mangiaterra G.; Cannalire R.; Cedraro N.; Pietrella D.; Astolfi A.; Massari S.; Tabarrini O.; Manfroni G.; Barreca M. L.; Cecchetti V.; Biavasco F.; Sabatini S. C-2 phenyl replacements to obtain potent quinoline-based Staphylococcus aureus NorA inhibitors. J. Enzyme Inhib Med. Chem. 2020, 35, 584–597. 10.1080/14756366.2020.1719083. 31992093 PMC7034129

[ref32] Felicetti T.; Machado D.; Cannalire R.; Astolfi A.; Massari S.; Tabarrini O.; Manfroni G.; Barreca M. L.; Cecchetti V.; Viveiros M.; Sabatini S. Modifications on C6 and C7 Positions of 3-Phenylquinolone Efflux Pump Inhibitors Led to Potent and Safe Antimycobacterial Treatment Adjuvants. ACS Infect Dis 2019, 5, 982–1000. 10.1021/acsinfecdis.9b00041. 30907573

[ref33] Cannalire R.; Mangiaterra G.; Felicetti T.; Astolfi A.; Cedraro N.; Massari S.; Manfroni G.; Tabarrini O.; Vaiasicca S.; Barreca M. L.; Cecchetti V.; Biavasco F.; Sabatini S. Structural Modifications of the Quinolin-4-yloxy Core to Obtain New Staphylococcus aureus NorA Inhibitors. Int. J. Mol. Sci. 2020, 21, 7037 10.3390/ijms21197037. 32987835 PMC7582826

[ref34] Palazzotti D.; Bissaro M.; Bolcato G.; Astolfi A.; Felicetti T.; Sabatini S.; Sturlese M.; Cecchetti V.; Barreca M. L.; Moro S. Deciphering the Molecular Recognition Mechanism of Multidrug Resistance Staphylococcus aureus NorA Efflux Pump Using a Supervised Molecular Dynamics Approach. Int. J. Mol. Sci. 2019, 20, 4041 10.3390/ijms20164041. 31430864 PMC6719125

[ref35] Felicetti T.; Cannalire R.; Pietrella D.; Latacz G.; Lubelska A.; Manfroni G.; Barreca M. L.; Massari S.; Tabarrini O.; Kiec-Kononowicz K.; Schindler B. D.; Kaatz G. W.; Cecchetti V.; Sabatini S. 2-Phenylquinoline S. aureus NorA Efflux Pump Inhibitors: Evaluation of the Importance of Methoxy Group Introduction. J. Med. Chem. 2018, 61, 7827–7848. 10.1021/acs.jmedchem.8b00791. 30067360

[ref36] Barreca M. L.; De Luca L.; Iraci N.; Chimirri A. Binding mode prediction of strand transfer HIV-1 integrase inhibitors using Tn5 transposase as a plausible surrogate model for HIV-1 integrase. J. Med. Chem. 2006, 49, 3994–3997. 10.1021/jm060323r. 16789757

[ref37] Ferro S.; De Luca L.; Barreca M. L.; Iraci N.; De Grazia S.; Christ F.; Witvrouw M.; Debyser Z.; Chimirri A. Docking studies on a new human immunodeficiency virus integrase-Mg-DNA complex: phenyl ring exploration and synthesis of 1H-benzylindole derivatives through fluorine substitutions. J. Med. Chem. 2009, 52, 569–573. 10.1021/jm8009266. 19105658

[ref38] Cuzzolin A.; Sturlese M.; Deganutti G.; Salmaso V.; Sabbadin D.; Ciancetta A.; Moro S. Deciphering the Complexity of Ligand-Protein Recognition Pathways Using Supervised Molecular Dynamics (SuMD) Simulations. J. Chem. Inf Model 2016, 56, 687–705. 10.1021/acs.jcim.5b00702. 27019343

[ref39] Salmaso V.; Moro S. Bridging Molecular Docking to Molecular Dynamics in Exploring Ligand-Protein Recognition Process: An Overview. Front Pharmacol 2018, 9, 923 10.3389/fphar.2018.00923. 30186166 PMC6113859

[ref40] Bissaro M.; Sturlese M.; Moro S. Exploring the RNA-Recognition Mechanism Using Supervised Molecular Dynamics (SuMD) Simulations: Toward a Rational Design for Ribonucleic-Targeting Molecules?. Front Chem. 2020, 8, 107 10.3389/fchem.2020.00107. 32175307 PMC7057144

[ref41] Pavan M.; Bolcato G.; Bassani D.; Sturlese M.; Moro S. Supervised Molecular Dynamics (SuMD) Insights into the mechanism of action of SARS-CoV-2 main protease inhibitor PF-07321332. J. Enzyme Inhib Med. Chem. 2021, 36, 1645–1649. 10.1080/14756366.2021.1954919. PMC8300928 34289752

[ref42] Salmaso V.; Sturlese M.; Cuzzolin A.; Moro S. Exploring Protein-Peptide Recognition Pathways Using a Supervised Molecular Dynamics Approach. Structure 2017, 25, 655–662. 10.1016/j.str.2017.02.009. 28319010

[ref43] Grosjean H.; Isik M.; Aimon A.; Mobley D.; Chodera J.; von Delft F.; Biggin P. C. SAMPL7 protein-ligand challenge: A community-wide evaluation of computational methods against fragment screening and pose-prediction. J. Comput. Aided Mol. Des 2022, 36, 291–311. 10.1007/s10822-022-00452-7. 35426591 PMC9010448

[ref44] Ferrari F.; Bissaro M.; Fabbian S.; De Almeida Roger J.; Mammi S.; Moro S.; Bellanda M.; Sturlese M. HT-SuMD: making molecular dynamics simulations suitable for fragment-based screening. A comparative study with NMR. J. Enzyme Inhib Med. Chem. 2021, 36, 1–14. 10.1080/14756366.2020.1838499. 33115279 PMC7598995

[ref45] Harvey M. J.; Giupponi G.; Fabritiis G. D. ACEMD: Accelerating Biomolecular Dynamics in the Microsecond Time Scale. J. Chem. Theory Comput 2009, 5, 1632–1639. 10.1021/ct9000685. 26609855

[ref46] Vanommeslaeghe K.; Hatcher E.; Acharya C.; Kundu S.; Zhong S.; Shim J.; Darian E.; Guvench O.; Lopes P.; Vorobyov I.; Mackerell A. D. Jr. CHARMM general force field: A force field for drug-like molecules compatible with the CHARMM all-atom additive biological force fields. J. Comput. Chem. 2009, 31, 671–690. 10.1002/jcc.21367. PMC2888302 19575467

[ref47] Berman H. M.; Westbrook J.; Feng Z.; Gilliland G.; Bhat T. N.; Weissig H.; Shindyalov I. N.; Bourne P. E. The Protein Data Bank. Nucleic Acids Res. 2000, 28, 235–242. 10.1093/nar/28.1.235. 10592235 PMC102472

[ref48] Schrödinger Release 2021–3: Protein Preparation Wizard; Epik; Impact; Prime; Schrödinger, LLC: New York, 2021.

[ref49] Schrödinger Release 2021–3: Epik; Schrödinger, LLC: New York, 2021.

[ref50] Schrödinger Release 2021–3: Prime; Schrödinger, LLC: New York, 2021.

[ref51] Rarey M.; Dixon J. S. Feature trees: a new molecular similarity measure based on tree matching. J. Comput. Aided Mol. Des 1998, 12, 471–490. 10.1023/A:1008068904628. 9834908

[ref52] Schrödinger Release 2021–3: LigPrep; Schrödinger, LLC: New York, 2021.

[ref53] Humphrey W.; Dalke A.; Schulten K. VMD: visual molecular dynamics. J. Mol. Graph 1996, 14 (1), 33–38. 10.1016/0263-7855(96)00018-5. 8744570

[ref54] Grubmuller H.; Groll V. Solvate 1.0; Max Planck Institute for Multidisciplinary Sciences.

[ref55] McGibbon R. T.; Beauchamp K. A.; Harrigan M. P.; Klein C.; Swails J. M.; Hernandez C. X.; Schwantes C. R.; Wang L. P.; Lane T. J.; Pande V. S. MDTraj: A Modern Open Library for the Analysis of Molecular Dynamics Trajectories. Biophys. J. 2015, 109, 1528–1532. 10.1016/j.bpj.2015.08.015. 26488642 PMC4623899

[ref56] Bakan A.; Meireles L. M.; Bahar I. ProDy: protein dynamics inferred from theory and experiments. Bioinformatics 2011, 27, 1575–1577. 10.1093/bioinformatics/btr168. 21471012 PMC3102222

[ref57] Ester M.; Kriegel H.-P.; Sander J.; Xu X. A density-based algorithm for discovering clusters in large spatial databases with noise. In Proceedings of the Second International Conference on Knowledge Discovery and Data Mining; AAAI Press: Portland, OR, 1996.

[ref58] Friesner R. A.; Banks J. L.; Murphy R. B.; Halgren T. A.; Klicic J. J.; Mainz D. T.; Repasky M. P.; Knoll E. H.; Shelley M.; Perry J. K.; Shaw D. E.; Francis P.; Shenkin P. S. Glide: a new approach for rapid, accurate docking and scoring. 1. Method and assessment of docking accuracy. J. Med. Chem. 2004, 47, 1739–1749. 10.1021/jm0306430. 15027865

[ref59] Halgren T. A.; Murphy R. B.; Friesner R. A.; Beard H. S.; Frye L. L.; Pollard W. T.; Banks J. L. Glide: a new approach for rapid, accurate docking and scoring. 2. Enrichment factors in database screening. J. Med. Chem. 2004, 47, 1750–1759. 10.1021/jm030644s. 15027866

[ref60] Deng Z.; Chuaqui C.; Singh J. Structural interaction fingerprint (SIFt): a novel method for analyzing three-dimensional protein-ligand binding interactions. J. Med. Chem. 2004, 47, 337–344. 10.1021/jm030331x. 14711306

[ref61] Bissaro M.; Bolcato G.; Pavan M.; Bassani D.; Sturlese M.; Moro S. Inspecting the Mechanism of Fragment Hits Binding on SARS-CoV-2 M(pro) by Using Supervised Molecular Dynamics (SuMD) Simulations. ChemMedChem. 2021, 16, 2075–2081. 10.1002/cmdc.202100156. 33797868 PMC8250706

[ref62] Hassankalhori M.; Bolcato G.; Bissaro M.; Sturlese M.; Moro S. Shedding Light on the Molecular Recognition of Sub-Kilodalton Macrocyclic Peptides on Thrombin by Supervised Molecular Dynamics. Front Mol. Biosci 2021, 8, 707661 10.3389/fmolb.2021.707661. 34532343 PMC8438215

[ref63] Astolfi A.; Felicetti T.; Iraci N.; Manfroni G.; Massari S.; Pietrella D.; Tabarrini O.; Kaatz G. W.; Barreca M. L.; Sabatini S.; Cecchetti V. Pharmacophore-Based Repositioning of Approved Drugs as Novel Staphylococcus aureus NorA Efflux Pump Inhibitors. J. Med. Chem. 2017, 60, 1598–1604. 10.1021/acs.jmedchem.6b01439. 28117588

[ref64] Milletti F.; Storchi L.; Sforna G.; Cruciani G. New and original pKa prediction method using grid molecular interaction fields. J. Chem. Inf Model 2007, 47, 2172–2181. 10.1021/ci700018y. 17910431

[ref65] Vazquez J.; Lopez M.; Gibert E.; Herrero E.; Luque F. J. Merging Ligand-Based and Structure-Based Methods in Drug Discovery: An Overview of Combined Virtual Screening Approaches. Molecules 2020, 25, 4723 10.3390/molecules25204723. 33076254 PMC7587536

[ref66] Wilson G. L.; Lill M. A. Integrating structure-based and ligand-based approaches for computational drug design. Future Med. Chem. 2011, 3, 735–750. 10.4155/fmc.11.18. 21554079

[ref67] Caroli A.; Ballante F.; Wickersham R. B. 3rd; Corelli F.; Ragno R. Hsp90 inhibitors, part 2: combining ligand-based and structure-based approaches for virtual screening application. J. Chem. Inf Model 2014, 54, 970–977. 10.1021/ci400760a. 24555544 PMC3985681

[ref68] Astolfi A.; Kudolo M.; Brea J.; Manni G.; Manfroni G.; Palazzotti D.; Sabatini S.; Cecchetti F.; Felicetti T.; Cannalire R.; Massari S.; Tabarrini O.; Loza M. I.; Fallarino F.; Cecchetti V.; Laufer S. A.; Barreca M. L. Discovery of potent p38alpha MAPK inhibitors through a funnel like workflow combining in silico screening and in vitro validation. Eur. J. Med. Chem. 2019, 182, 111624 10.1016/j.ejmech.2019.111624. 31445234

