# Supplementary material for: Fighting Antimicrobial Resistance: Insights on How the Staphylococcus aureus NorA Efflux Pump Recognizes 2-Phenylquinoline Inhibitors by Supervised Molecular Dynamics (SuMD) and Molecular Docking Simulations
Source: J Chem Inf Model. 2023 Jul 29;63(15):4875–87. doi: 10.1021/acs.jcim.3c00516 (PMC10428217; doi:10.1021/acs.jcim.3c00516)
Supplement: Supplementary file 1 — ci3c00516_si_001.pdf [file ci3c00516_si_001.pdf]

Supporting Information

Fighting antimicrobial resistance: insights on how the S. aureus NorA Efflux Pump Recognizes 2-phenylquinoline Inhibitors by Supervised Molecular Dynamics (SuMD) and Molecular Docking Simulations

Deborah Palazzotti, [1] Tommaso Felicetti, [1] Stefano Sabatini, [1] Stefano Moro, [2] Maria Letizia Barreca, [1] Mattia Sturlese [2]* and Andrea Astolfi [1]*.

[1]Department of Pharmaceutical Sciences, "Department of Excellence 2018-2022", University of Perugia, Via del Liceo, 1, 06123, Perugia, Italy.

[2]Molecular Modeling Section (MMS), Department of Pharmaceutical and Pharmacological Sciences, University of Padova, via Marzolo 5, 35131 Padova, Italy

To whom corresponding should be addressed: mattia.sturlese@unipd.it, andrea.astolfi@unipg.it

Table of Contents

1. **Supporting Figures and Tables**

1 Supporting Figures and Tables

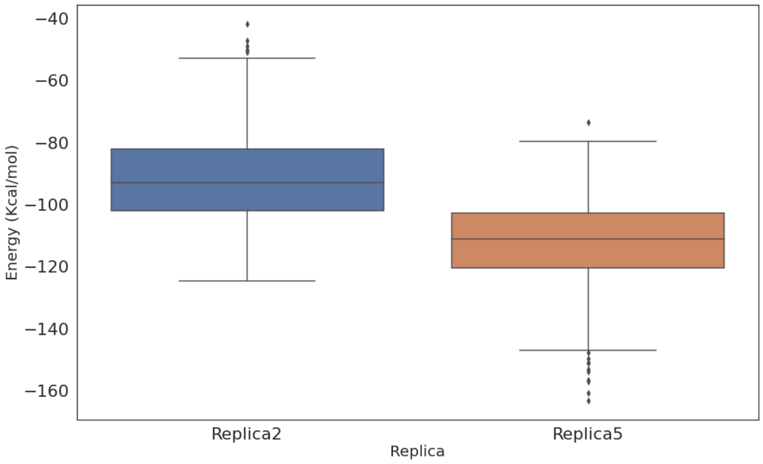

Figure S1. Boxplot of the energy profile for replica 2 and replica 5.

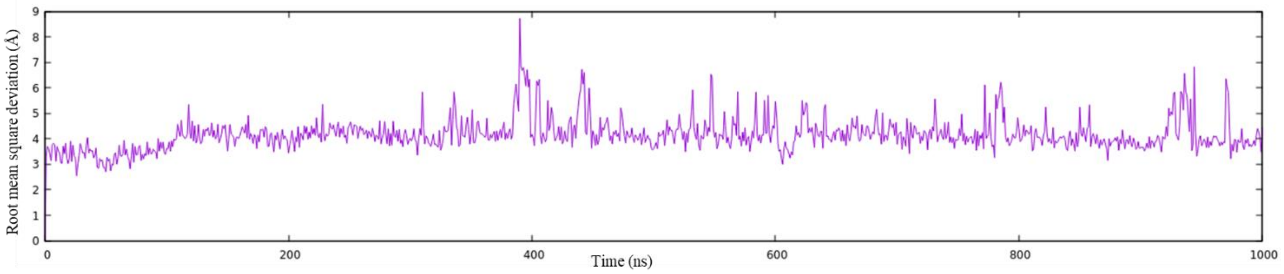

Figure S2. PQQ16P root mean square deviation obtained for the 1 µs long MD simulation. The starting point of the MD was the **PQQ16P** binding mode obtained in SuMD replica 5.

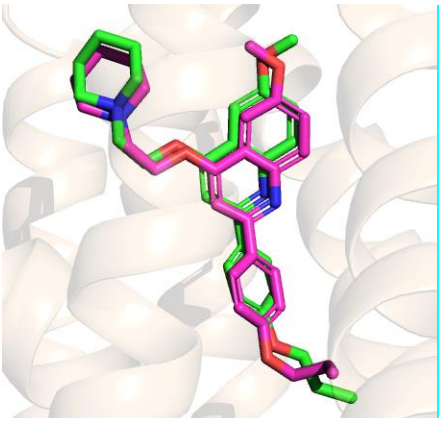

Figure S3. Superimposition between SuMD (green) and Glide XP protocol (magenta) predicted binding modes of **PQQ16P.**

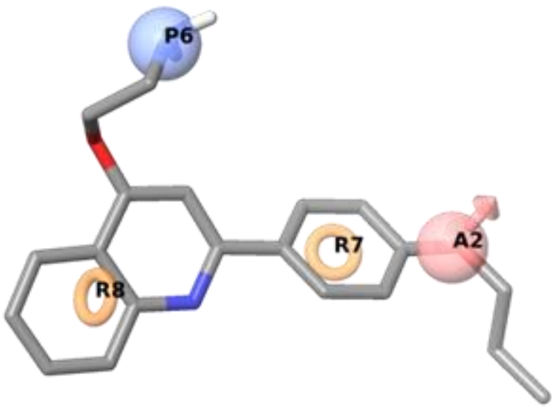

Figure S4. Common feature pharmacophore model generated in our previous work. It consists of one hydrogenbond acceptor (A2), one positive charge (P6), and two aromatic rings (R7 and R8).

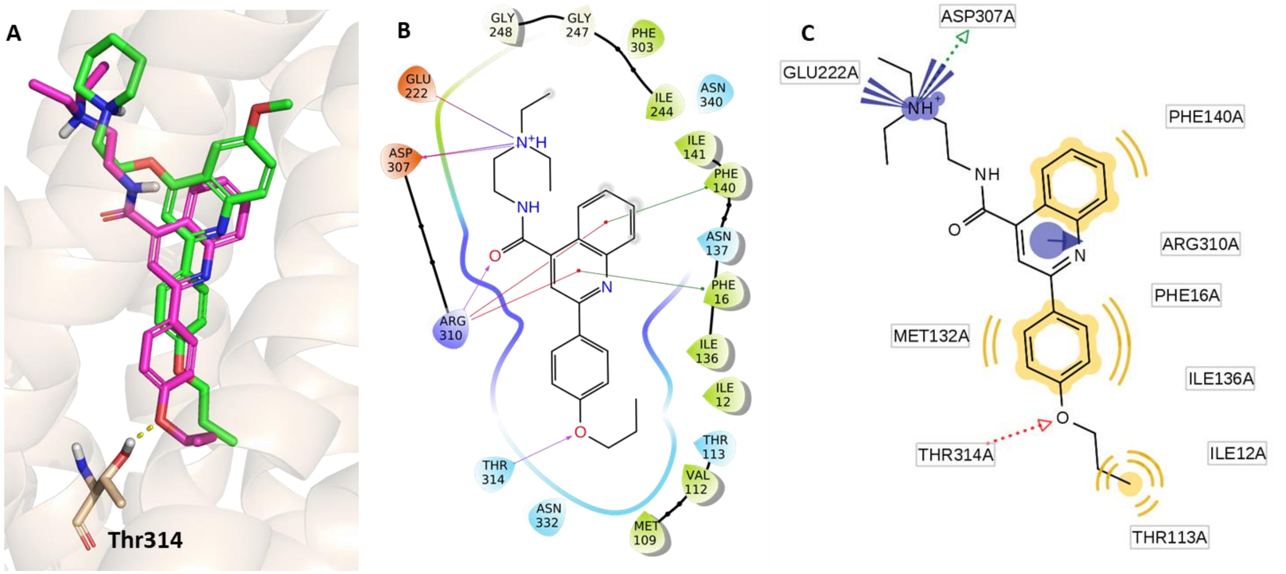

Figure S5: Representation of **7** binding mode into NorA efflux pump. A) Superimposition between the **PQQ16P** SuMD-derived pose (green) and Glide XP (magenta) predicted binding mode of **7**. 2D schematic representation of key intermolecular interactions as suggested by the Ligand Interaction tool of Maestro (B) and LigandScout (C).

Table S1. Number of short unbiased MD simulations ran for each replica

**Replica**	**Number of short unbiased MD simulations**	
	Total	***To reach the site***
**1**	85	*NA*
**2**	85	70
**3**	66	*NA*
**4**	45	*NA*
**5**	43	15
**6**	85	*NA*
**7**	85	*NA*
NA: Not aplicable		

Table S2. NorA active and inactive sets. For each compound the binding pose cluster computed by SFPt analysis, the percentage of EtBr efflux inhibition at 50 μM (%EtBr inh), the computed MMGBSA ΔG of binding (MMGBSA dG Bind) and the smile structure are reported.

Cpd	binding pose cluster	%EtBr inh.	MMGBSA dG Bind	SMILE
**1**	**12**	100	-67.51	CN(C)CCCOc1cc(nc(c12)cc(cc2)OC)-c3ccc(cc3)OCCC
**2**	**12**	100	-66.26	CCCOc(cc1)ccc1-c(nc(c23)cc(cc3)OC)cc2OCCN(CC)CC
**3**	**12**	100	-71.43	CCCOc(cc1)ccc1-c(nc(c23)cc(cc3)OC)cc2OCCN4CCCCC4
**4**	4	100	-68.73	CCCOc(cc1)ccc1-c(nc(c23)cc(cc3)OC)cc2OCCN4CCNCC4
**5**	**12**	100	-69.33	CCCOc(cc1)ccc1-c(nc(c23)c(OC)ccc3)cc2OCCN4CCNCC4
**6**	**12**	99	-80.46	CCCOc(cc1)ccc1-c(nc(c23)ccc(c3)OC)cc2OCCN(CC)CC
**7**	**12**	98.1	-62.89	CCN(CC)CCNC(=O)c1cc(nc(c12)cccc2)-c3ccc(cc3)OCCC
**PQQ16P**	**12**	97.6	-73.58	CCCOc(cc1)ccc1-c(nc(c23)ccc(c3)OC)cc2OCCN4CCCCC4
**8**	**12**	96.5	-68.86	CCCOc(cc1)ccc1-c(cc2OCCN(CC)CC)nc(c23)cccc3OC
**9**	**12**	96.4	-73.13	C1CCCCN1CCNC(=O)c2cc(nc(c23)cccc3)-c4ccc(cc4)OCCC
**10**	**12**	96.4	-62.26	CCCOc(cc1)ccc1-c(cc2OCCN(C)C)nc(c23)cc(OC)c(c3)OC
**11**	**12**	96.2	-66.86	CCCOc(cc1)ccc1-c(nc(c23)cc(OC)c(c3)OC)cc2OCCN4CCCC4
**12**	**12**	96.1	-52.81	CCCOc(cc1)ccc1-c(cc2OCCN)nc(c23)cccc3
**13**	**12**	95.2	-70.47	CCCOc(cc1)ccc1-c(nc(c23)cc(OC)c(c3)OC)cc2OCCN(CC)CC
**14**	**12**	95	-63.32	CCCOc(cc1)ccc1-c(nc(c23)ccc(c3)O)cc2OCCN(CC)CC
**15**	11	94.2	-68.51	CCCOc(cc1)ccc1-c(nc(c23)cccc3)cc2OCCN4CCCC4
**16**	**12**	94.1	-63.22	CCCOc(cc1)ccc1-c(nc(c23)cc(OC)c(c3)OC)cc2OCCN(CC4)Cc(c45)cc(OC)c(c5)OC
**17**	**12**	94.1	-61.67	CCCOc(cc1)ccc1-c(nc(c23)c(OC)cc(c3)OC)cc2OCCN4CCCCC4
**18**	11	93.6	-45.65	CCCOc(cc1)ccc1-c(cc2OCCN(C)C)nc(c23)cc(OC)cc3OC
**19**	**12**	93.4	-65.52	CCCOc(cc1)ccc1-c(nc(c23)cccc3)cc2OCCN(CC)CC
**20**	**12**	93.4	-59.17	CCCOc(cc1)ccc1-c(nc(c23)cccc3)cc2OCCN4CCNCC4
**21**	13	93.2	-73.88	C1COCCN1CCCOc(c2)ccc(c23)nc(-c4ccc(cc4)OCCC)cc3OCCN5CCCCC5
**22**	**12**	93.1	-65.72	CCCOc(cc1)ccc1-c(cc2OCCN(CC)CC)nc(c23)cc(OC)cc3OC
**23**	**12**	92.5	-73.24	CN(C)CCCOc(c1)ccc(c12)nc(-c3ccc(cc3)OCCC)cc2OCCN4CCCCC4
**24**	11	92.3	-64.01	CCCOc(cc1)ccc1-c(nc(c23)c(OC)cc(c3)OC)cc2OCCN(CC)CC
**25**	**12**	92.2	-68	CCCOc(cc1)ccc1-c(nc(c23)ccc(c3)OC)cc2OCCN4CCCCCC4
**26**	**12**	92.1	-70.46	CCCOc(cc1)ccc1-c(nc(c23)cc(OC)c(c3)OC)cc2OCCN4CCCCC4
**27**	**12**	92.1	-81.79	CCCOc(cc1)ccc1-c(nc(c23)cc(cc3)OC)cc2OCCN4CCN(CC4)Cc5ccccc5
**28**	11	92	-49.05	CCCOc(cc1)ccc1-c(nc(c23)cccc3)cc2OCCN(C)C
**29**	**12**	91.6	-52.09	CCCOc(cc1)ccc1Cc(n2)n(c(c23)cccc3)CCN4CCCCC4
**30**	**12**	91.4	-74.08	C1CCCCN1CCOc2cc(-c3ccc(cc3)OCCC)nc(c24)ccc(c4)OCCN(CC)CC
**31**	**12**	91.3	-62.96	CCCOc(cc1)ccc1-c(nc(c23)cc(cc3)OC)cc2OCCN4CCCCCC4
**32**	**12**	91.1	-62.77	CCCOc(cc1)ccc1-c(cc2OCCN(CC)CC)nc(c23)ccc(c3)OCc4ccccc4
**33**	**12**	91.1	-57.49	C1COCCN1CCCOc(c2)ccc(c23)nc(-c4ccc(cc4)OCCC)cc3OCCN(CC)CC

Table S1. Continued.

Cpd	binding pose cluster	%EtBr inh.	MMGBSA dG Bind	SMILE
**34**	**12**	91	-54.37	CCCOc(cc1)ccc1-c(nc(c23)cccc3)cc2OCCN4CCN(C)CC4
**35**	**12**	91	-77.87	CCCOc(cc1)ccc1-c(nc(c23)cc(cc3)OC)cc2OCCN(CC4)Cc(c45)cc(OC)c(c5)OC
**36**	6	90.6	-61.73	CN(C)CCCOc1cc(nc(c12)cccc2)-c3ccc(cc3)OCCC
**37**	**12**	90.2	-54.74	CCCOc(cc1)ccc1-c(nc(c23)cccc3)nc2OCCN(C)C
**40**	9	29.5	-46.68	CCCOc(cc1)ccc1-c(n2)cc(=O)n(c23)cccc3
**41**	11	28.9	-38.35	CCCOc(cc1)ccc1-c(n(c2=O)CCN(C)C)nc(c23)cccc3
**42**	15	22.4	-47.86	CCCOc(cc1)ccc1-c(nc(c23)cccc3)n(c2=O)CCN(CC)CC
**43**	9	22.1	-47.22	CCCOc(cc1)ccc1C(=N2)COc(c23)cccc3
**44**	9	19.8	-68.29	C1CCCCN1CCOc(cc2)ccc2-c(cc3=O)n(C)c(c34)cccc4
**45**	4	19.6	-68.55	CCCOc(cc1)ccc1-c(nc(c23)cccc3)cc2OCc4ccccn4
**46**	9	16.3	-38.9	COc(cc1)ccc1C(=CC2)Nc(c23)cccc3
**47**	9	16.2	-56.26	COc1c(OC)cc(cc1OC)[C@@H](CC2)Nc(c23)cccc3
**48**	9	16.2	-44.33	COc1c(OC)cc(cc1OC)[C@H](CC2)Nc(c23)cccc3
**49**	2	15.2	-39.6	c1cccc(c12)N[C@H](CC2)c3ccccc3
**50**	9	15.2	-41.51	c1cccc(c12)N[C@@H](CC2)c3ccccc3
**51**	3	14.5	-46.42	CCCOc(cc1)ccc1C(=CC2)Nc(c23)cccc3
**52**	9	13.3	-58.84	c1ccc(OC)c(c12)nc(cc2OC=C)-c3ccc(cc3)OCCC
**53**	9	12.5	-38.22	CCCOc(cc1)ccc1-c(n2)cnc(c23)cccc3
**54**	9	10.8	-48.87	CCOc(cc1)ccc1-c(cc2=O)n(C)c(c23)cccc3
**55**	9	10.5	-58.67	CCCOc(cc1)ccc1-c(cc2OCCCl)nc(c23)ccc(c3)OC
**56**	9	10.3	-46.93	CC(C)Oc(cc1)ccc1-c(cc2=O)n(C)c(c23)cccc3
**57**	9	9.4	-51.46	COc(c1)c(OC)cc(c12)n(C)c(cc2=O)-c3ccccc3
**58**	6	9.1	-46.73	CCCOc(cc1)ccc1-c(cc2=O)n(C)c(c23)cc(O)c(c3)OC
**59**	9	9.1	-47.53	CCCOc(cc1)ccc1Cc(cc2=O)[nH]c(c23)cccc3
**60**	9	8.6	-47.76	COc1c(OC)ccc(c1)[C@@H](CC2)Nc(c23)cccc3
**61**	9	8.6	-40.91	COc1c(OC)ccc(c1)[C@H](CC2)Nc(c23)cccc3
**62**	10	8.3	-69.65	CCCOc(cc1)ccc1-c(nc(c23)cccc3)cc2OCCN(C4=O)C(=O)c(c45)cccc5
**63**	9	8.1	-57.86	COc1c(OC)cc(cc1OC)-c(n2)cnc(c23)cccc3
**64**	9	8.1	-39.44	COc1c(OC)cc(cc1OC)[C@@H](N2)CNc(c23)cccc3
**65**	9	8.1	-54.58	COc1c(OC)cc(cc1OC)[C@H](N2)CNc(c23)cccc3
**66**	9	6.8	-58.63	CCCOc(cc1)ccc1-c(cc2OCCCl)nc(c23)c(OC)ccc3
**67**	9	5.8	-40.61	COc(cc1)ccc1[C@@H](CC2)Nc(c23)cccc3
**68**	9	5.8	-39.87	COc(cc1)ccc1[C@H](CC2)Nc(c23)cccc3
**69**	9	4.8	-30.63	CCCOc(cc1)ccc1-c(cc2=O)n(C)c(c23)cc(OC)cc3O
**70**	9	4.4	-55.01	CN(C)CCOc(cc1)ccc1-c(cc2=O)n(C)c(c23)cccc3
**71**	4	4.1	-56.89	CCCOc(cc1)ccc1-c(nc(c23)cccc3)cc2OCCn4cccc4
**72**	9	2.4	-50.33	COc(cc1)c(OC)cc1C(=N2)COc(c23)cccc3
**73**	6	1.7	-60.89	CCN(CC)CCOc(cc1)ccc1-c(cc2=O)n(C)c(c23)cccc3
**38**	**12**	0	-46.05	c1c(O)c(O)cc(c12)n(C)c(cc2=O)-c3ccccc3
**39**	**12**	0	-76.18	CCCOc(cc1)ccc1-c(nc(c23)cccc3)cc2OCCN(Cc4ccccc4)Cc5ccccc5

Table S1. Continued.

Cpd	binding pose cluster	%EtBr inh.	MMGBSA dG Bind	SMILE
**74**	15	0	-55.52	O=C(O)c(c1=O)cn(C2CC2)c(c13)cc(c(c3)N)-n(ccc4=O)c(c45)cccc5
**75**	14	0	-50.35	CCCOc(cc1)ccc1-c([nH]c2=O)nc(c23)cccc3
**76**	9	0	-44.72	CCCOc(cc1)ccc1-c(cc2=O)[nH]c(c23)cccc3
**77**	14	0	-46.28	CCCOc(cc1)ccc1-c(cc2=O)[nH]c(c23)cc(OC)c(c3)OC
**78**	8	0	-49.41	CCCOc(cc1)ccc1-c(cc2=O)n(C)c(c23)cc(OC)c(c3)OC
**79**	2	0	-54.87	c1cccc(c12)n(C)c(cc2=O)-c3ccc(O)cc3
**80**	9	0	-41.68	c1cccc(c12)n(C)c(cc2=O)-c3ccc(cc3)OC
**81**	8	0	-49.8	COc(c1)c(OC)cc(c12)n(C)c(cc2=O)-c3ccc(O)cc3
**82**	7	0	-42.41	c1c(O)c(O)cc(c12)n(C)c(cc2=O)-c3ccc(O)cc3
**83**	9	0	-58.98	C1COCCN1CCOc(cc2)ccc2-c(cc3=O)n(C)c(c34)cccc4
**84**	2	0	-40.48	c1ccccc1C(=N2)COc(c23)cccc3
**85**	9	0	-37.58	c1cccc(c12)OC[C@H](N2)c3ccccc3
**86**	2	0	-54.75	c1cccc(c12)OC[C@@H](N2)c3ccccc3
**87**	9	0	-42.19	COc(cc1)ccc1C(=N2)COc(c23)cccc3
**88**	9	0	-41.13	COc(cc1)ccc1[C@@H](N2)COc(c23)cccc3
**89**	9	0	-41.53	COc(cc1)ccc1[C@H](N2)COc(c23)cccc3
**90**	9	0	-43.09	COc1c(OC)ccc(c1)[C@@H](N2)COc(c23)cccc3
**91**	1	0	-48.92	COc1c(OC)ccc(c1)[C@H](N2)COc(c23)cccc3
**92**	9	0	-42.87	COc1c(OC)cc(cc1OC)[C@@H](N2)COc(c23)cccc3
**93**	9	0	-54.14	COc1c(OC)cc(cc1OC)[C@H](N2)COc(c23)cccc3
**94**	2	0	-40.52	c1ccccc1-c(n2)cnc(c23)cccc3
**95**	9	0	-48.08	COc(cc1)c(OC)cc1-c(n2)cnc(c23)cccc3
**96**	9	0	-36.58	COc1c(OC)ccc(c1)[C@@H](N2)CNc(c23)cccc3
**97**	9	0	-39.21	COc1c(OC)ccc(c1)[C@H](N2)CNc(c23)cccc3
**98**	11	0	-40.45	FC(F)(F)c(c1)ccc(c12)ncc(n2)-c3ccc([N+]([O-])=O)cc3
**99**	5	0	-44.65	FC(F)(F)c(c1)ccc(c12)nc(cn2)-c3ccc([N+]([O-])=O)cc3
**100**	8	0	-39.42	CCCOc(cc1)ccc1[C@@H](N2)CNc(c23)cccc3
**101**	1	0	-48.68	CCCOc(cc1)ccc1[C@H](N2)CNc(c23)cccc3
**102**	10	0	-41.07	CCCOc(cc1)ccc1-c(c2=O)c[nH]c(c23)cccc3
